# Cross-Platform Comparison of Multispectral and Time-Resolved Singlet Oxygen Luminescence Dosimetry Under Matched Irradiation Conditions in a Liquid Protoporphyrin IX Phantom

**DOI:** 10.3390/antiox15101250

**Published:** 2026-09-29

**Authors:** Baozhu Lu, Vikas Vikas, Weibing Yang, Robert H. Hadfield, Brian C. Wilson, Timothy C. Zhu

**Affiliations:** 1 Department of Radiation Oncology, University of Pennsylvania, Philadelphia, PA 19104, USA; baozhu.lu@pennmedicine.upenn.edu (B.L.); weibing.yang@pennmedicine.upenn.edu (W.Y.); 2 James Watt School of Engineering, University of Glasgow, Glasgow G128LT, UK; vikas.vikas.2@glasgow.ac.uk (V.V.); robert.hadfield@glasgow.ac.uk (R.H.H.); 3 Department of Medical Biophysics, University Health Network, University of Toronto, Toronto, ON M5G 1L7, Canada; brian.wilson@uhn.ca

**Keywords:** photodynamic therapy, singlet oxygen, dosimetry, MSOLD, TSOLD, SOED, PpIX, time correlated single photon counting

## Abstract

Accurate quantification of singlet oxygen (^1^O_2_) production is central to photodynamic therapy (PDT) dosimetry, yet direct measurement remains challenging due to the weak and transient nature of ^1^O_2_ luminescence. To our knowledge, this study presents the first controlled exploratory cross-platform comparison of Multispectral Singlet Oxygen Luminescence Dosimetry (MSOLD) and Time-Resolved Singlet Oxygen Luminescence Detection (TSOLD) under matched irradiation conditions. MSOLD enables continuous monitoring during irradiation through spectral decomposition of near-infrared emission, whereas TSOLD uses pulsed excitation and time-correlated single-photon counting to provide lifetime-resolved ^1^O_2_ measurements. Across PpIX concentrations of 10–100 mg/kg, both methods detected irradiation-induced decreases in ^1^O_2_ luminescence, while TSOLD provided stable lifetime estimates of approximately 10 µs in the methanol-rich phantom. Across fluence rates of 1–5 W/cm^2^, both techniques showed progressively larger irradiation-induced reductions in ^1^O_2_ luminescence with increasing irradiance. Cumulative MSOLD-derived ^1^O_2_ signals showed an approximately linear relationship with Singlet Oxygen Explicit Dosimetry (SOED) calculations (R^2^ > 0.98) under the investigated conditions. Together, these results demonstrate consistent concentration- and fluence-rate-dependent trends between MSOLD and TSOLD and highlight their complementary measurement characteristics: MSOLD provides continuous spectral monitoring during irradiation, whereas TSOLD provides lifetime-resolved characterization of ^1^O_2_ and photosensitizer kinetics. These findings provide a basis for future replicated and biologically relevant studies of luminescence-based PDT dosimetry.

## 1. Introduction

Singlet oxygen (^1^O_2_) is the primary cytotoxic species responsible for cellular damage during Type II photodynamic therapy (PDT) [1,2,3,4,5,6,7,8,9]. As a short-lived and highly reactive molecule, ^1^O_2_ provides a direct photochemical marker of PDT efficacy [5,10,11,12,13]. However, its near-infrared luminescence at ~1270 nm is intrinsically weak and rapidly quenched in biological media, making real-time detection technically challenging [3,5,14]. The resulting low signal-to-noise ratio necessitates highly sensitive optical detection systems and sophisticated signal extraction methods [14,15]. These limitations have long made the direct detection of ^1^O_2_ the “holy grail” of PDT dosimetry, as emphasized in several recent studies on multispectral and time-resolved luminescence detection techniques [5,6,16].

To address these challenges, two complementary optical dosimetry approaches have emerged. Multispectral Singlet Oxygen Luminescence Dosimetry (MSOLD) decomposes near-infrared emission spectra into constituent components—direct light, photosensitizer fluorescence, and the weak ^1^O_2_ luminescence—using spectral fitting or singular value decomposition methods (SVD) [5,16]. MSOLD enables continuous, real-time monitoring of singlet oxygen generation during PDT and has been validated in multiple phantom and tissue studies [4,16]. Recent work demonstrates that MSOLD accuracy is strongly influenced by tissue optical properties, including absorption and scattering, which modulate both treatment light distribution and luminescence collection efficiency [17]. Corrections for these optical property variations have been shown to significantly improve quantitative fidelity, supporting MSOLD’s potential for clinical PDT dosimetry [16].

In contrast, Time-Resolved Singlet Oxygen Luminescence Detection (TSOLD) isolates ^1^O_2_ emission through its characteristic microsecond-scale lifetime using pulsed excitation and time-correlated single-photon counting (TCSPC) [13,15,18]. Because lifetime-resolved detection discriminates ^1^O_2_ from background fluorescence and scattered light, TSOLD offers high specificity and can simultaneously extract singlet oxygen and photosensitizer triplet state lifetimes [3,4,5,13,18,19]. This approach has been central to advances in time-resolved SOLD instrumentation, where optimized optics and photon collection schemes have improved detector efficiency and reduced background interference [14,15]. Despite these advantages, TSOLD typically requires long acquisition times and high photon counts, limiting its performance at low photosensitizer concentrations or in highly attenuating media [3,4,5].

Previous studies have investigated both spectrally resolved and time-resolved approaches to singlet-oxygen luminescence detection, including implementations combining spectral and temporal discrimination. Time-resolved singlet oxygen measurements have also been performed at multiple detection wavelengths around the 1270 nm emission band to distinguish the characteristic singlet oxygen phosphorescence from other near-infrared emission contributions [20,21]. However, to our knowledge, a controlled side-by-side comparison of a continuous-wave multispectral spectrometer-based MSOLD system and a pulsed time-resolved TSOLD system under matched irradiation conditions has not been reported yet. Prior MSOLD studies have primarily focused on spectral signal extraction and correction for optical-property variations, whereas TSOLD studies have emphasized temporal discrimination and lifetime measurements [5,9,13,15,16,17,18,19,20,21]. A direct comparison under matched experimental conditions may therefore help clarify the complementary measurement characteristics of these approaches.

In the present exploratory study, MSOLD and TSOLD were compared under matched experimental conditions using a controlled solution-based scattering phantom containing approximately 97% methanol and 0.6% Intralipid, with one measurement performed by each system for every experimental condition. MSOLD data were acquired continuously throughout irradiation, whereas TSOLD measurements were obtained immediately before and after irradiation. By systematically varying photosensitizer concentration and irradiation fluence rate, irradiation-induced changes in ^1^O_2_ luminescence were evaluated using both systems, together with continuous and cumulative MSOLD measurements. MSOLD measurements are further compared with singlet oxygen explicit dosimetry (SOED) calculations [5,22,23,24,25] using a model-based comparison. These experiments provide an exploratory assessment of the consistency, measurement characteristics, and complementary capabilities of MSOLD and TSOLD and establish a basis for future replicated studies of luminescence-based PDT dosimetry.

## 2. Materials and Methods

### 2.1. Phantom Preparation

Liquid phantoms were prepared as controlled solution-based scattering systems for ^1^O_2_ measurements but were not intended to serve as direct surrogates for in vivo tissue. Protoporphyrin IX (PpIX; Sigma-Aldrich, St. Louis, MO, USA) was dissolved in dimethyl sulfoxide (DMSO) to prepare a 10 mg/mL stock solution. PpIX is used widely as a clinical PDT photosensitizer through its endogenous synthesis upon administration of the precursor aminolaevulinic acid [26]. A scattering phantom was prepared separately by diluting Intralipid in methanol to a final Intralipid concentration of 0.6% (*v*/*v*), corresponding to a reduced scattering coefficient of $\mu_{s}^{\prime }$ ≈ 9 cm^−1^ at 630 nm. The reduced scattering coefficient was determined experimentally using a broad-beam attenuation measurement. The phantom dimensions were sufficiently large relative to the measurement region that boundary effects were considered negligible, allowing the medium to be approximated as infinite for the optical-property analysis. Light intensity was measured as a function of propagation distance through the phantom and fitted to obtain the effective attenuation coefficient, *μ_eff_*. With the absorption coefficient, *μ_a_*, determined from the known concentration of India ink added to the measurement phantom, $\mu_{s}^{\prime }$ was calculated from *μ_eff_* using the diffusion approximation. Appropriate volumes of the PpIX stock solution were then added to achieve the desired PpIX concentrations. A fresh phantom was prepared independently for each PpIX concentration and fluence-rate condition. The samples were transferred to screw-capped rectangular optical-glass cuvettes (MSE Supplies LLC, Tucson, AZ, USA) with a 10 mm optical path length and a nominal volume of 3.5 mL. Excluding the negligible volume of the DMSO-based PpIX stock solution, the final phantom consisted primarily of methanol (~97% *v*/*v*), with commercial 20% Intralipid (Nutrilipid^®^ 20% I.V. Fat Emulsion, B. Braun Medical Inc., Bethlehem, PA, USA) added to yield a final Intralipid concentration of approximately 0.6%. Methanol was selected as the primary solvent because it provided favorable conditions for comparison of TSOLD and MSOLD measurements, while the added Intralipid introduced optical scattering to provide a controlled scattering environment for the measurements. Because methanol constituted approximately 97% of the phantom volume, the measured singlet-oxygen signal predominantly reflected the methanol-based environment. During measurements, the detection fiber was positioned directly against the cuvette surface to maximize collection efficiency and maintain a consistent geometry across experiments.

Experiments were conducted under two controlled conditions to evaluate ^1^O_2_ production. In the first set, PpIX concentrations of 10, 25, 50, 75, and 100 mg/kg were evaluated at a constant irradiation fluence rate of 5 W/cm^2^. These concentrations correspond to 17.8, 44.4, 88.9, 133.3, and 177.7 μmol/kg, respectively, based on the molecular weight of PpIX (562.66 g/mol). For comparison with volume-based molar concentration units, they correspond to approximately 14.2, 35.5, 70.9, 106.4, and 141.8 μM, respectively, after accounting for the estimated phantom density of 0.798 kg/L. PpIX concentrations are reported in mg/kg throughout the manuscript. In a second set, the PpIX concentration was fixed at 50 mg/kg while illumination fluence rates were varied from 1 to 5 W/cm^2^ in increments of 1 W/cm^2^. For all conditions, photosensitizer concentrations were measured before and after irradiation to evaluate photobleaching and the associated changes in ^1^O_2_ production.

### 2.2. Phantom Irradiation

Phantoms were irradiated using a 630 nm diode laser (Ceralas, biolitec/CeramOptec, Bonn, Germany), matched to the absorption peak of PpIX. Light delivery was achieved via an optical fiber (Pioneer Optics, East Granby, CT, USA) terminated with a micro-lens to produce uniform illumination within the target volume.

A short-pass filter (FESH0800; Thorlabs, Newton, NJ, USA; cut-off wavelength: 800 nm) was placed in the excitation path to suppress long-wavelength components. Each sample underwent 900 s (15 min) of irradiation and delivered fluence rates were recorded for all experimental conditions. For the PpIX concentration-dependent experiments, the fluence rate was maintained at 5 W/cm^2^, whereas for the fluence-rate-dependent experiments, the PpIX concentration was held constant, and the fluence rate was varied from 1 to 5 W/cm^2^. For each experimental condition, the measurement sequence was (1) pre-irradiation TSOLD acquisition; (2) 900 s of 630 nm irradiation with continuous MSOLD acquisition; and (3) post-irradiation TSOLD acquisition. The same phantom was used throughout this sequence, with no replacement by a newly prepared phantom between the pre- and post-irradiation measurements. TSOLD measurements were therefore obtained immediately before and after irradiation under unchanged detection conditions, whereas MSOLD signals were recorded continuously during irradiation and reflected the temporal evolution of the ^1^O_2_ luminescence signal during irradiation.

Although the fluence rates used in the present phantom experiments were substantially higher than those typically used for clinical PDT, this choice was made to generate a sufficiently large and measurable change in the TSOLD signal over the 900 s irradiation period, thereby enabling a clear comparison with the dynamic MSOLD measurements. Importantly, our previous in vivo study demonstrated that the same TSOLD system could directly detect ^1^O_2_ luminescence from RIF tumors during Photofrin-mediated PDT at a clinically relevant fluence rate of 100 mW/cm^2^ [15]. Therefore, these higher fluence rates were selected to produce a measurable pre- to post-irradiation signal change and a sufficiently broad signal range for comparison of TSOLD and MSOLD under the present phantom conditions and should not be interpreted as a formal assessment of TSOLD detection sensitivity.

### 2.3. Multispectral Singlet Oxygen Luminescence Dosimetry (MSOLD)

**Figure 1:**
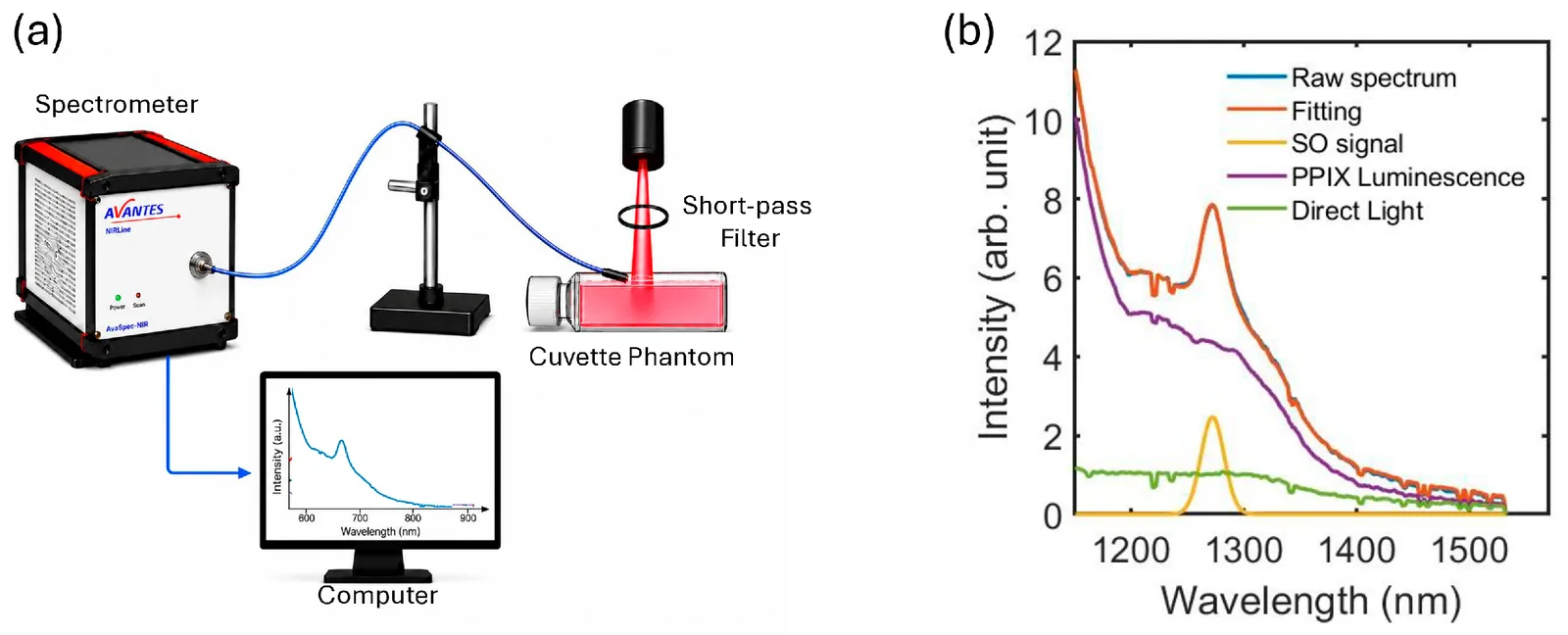
MSOLD acquisition setup and spectral decomposition. (**a**) Schematic of the multispectral singlet oxygen luminescence dosimetry (MSOLD) data acquisition setup. (**b**) Representative MSOLD spectrum decomposed into direct light, protoporphyrin IX (PpIX) luminescence, and singlet oxygen components, with fitted spectrum overlaid on the measured spectrum.

MSOLD measurements shown in Figure 1a were performed using a high-OH, SMA-to-SMA fiber patch cable (M93L; Thorlabs, Newton, NJ, USA) with a 1500 ± 30 μm core diameter and a numerical aperture of 0.39 for signal collection. The collected emission was coupled to a thermoelectrically cooled near-infrared spectrometer (NIR256-1.7-HSC-EVO, Avantes, Apeldoorn, The Netherlands), operated in high-sensitivity (HS) mode. According to the manufacturer, the HS mode provides a sensitivity of approximately 4.8 × 10^6^ counts/µW per ms over the 1000–1750 nm range, with a specified signal-to-noise ratio of approximately 1900:1 and a dynamic range of approximately 4900:1. The spectrometer provided a wavelength sampling interval of approximately 1.51 nm per detector pixel. These specifications reflect detector performance under standardized conditions and are distinct from the experimentally determined SNR of the extracted ^1^O_2_ signal. Spectra were continuously acquired during irradiation using an integration time of 800 ms, with the collection fiber maintained in a fixed position directly against the cuvette surface to minimize geometry-dependent variations.

The output from the micro-lens-coupled light source (Pioneer P/N 5721, Pioneer Optics Company, East Granby, CT, USA) was spectrally cleaned using an 800 nm short-pass filter (FESH0800; Thorlabs) before illuminating an approximately 6 mm-diameter irradiation area on the phantom.

To isolate the ^1^O_2_ phosphorescence near 1270 nm, the measured spectra were decomposed using singular value decomposition (SVD) into contributions from direct excitation light, photosensitizer luminescence, and ^1^O_2_ emission. The direct excitation basis was characterized by using phantoms without photosensitizer. The spectrum used to derive the PpIX luminescence basis was acquired from a 5 mL phantom containing 25 mg/kg PpIX under an irradiation fluence rate of 550 mW/cm^2^. Sodium azide was prepared in DI water at 5 mg/mL, and 300 µL was added to the phantom, resulting in a final concentration of approximately 4.35 mM. Additional sodium azide was subsequently added, and no further change in the measured spectrum was observed, confirming effective quenching of the ^1^O_2_ luminescence under these experimental conditions. Comparison of the spectra before and after sodium-azide quenching isolated the ^1^O_2_ emission component and showed that its spectral peak was centered at approximately 1273 nm. The direct-light component was obtained by directing the excitation light into the detection system using a phantom without PpIX. The PpIX luminescence component was then derived by subtracting the direct-light component from the spectrum measured after ^1^O_2_ quenching with sodium azide. The direct-light, PpIX luminescence, and ^1^O_2_ spectral components were individually normalized and used as basis spectra for SVD fitting in MATLAB R2024a. Because the scattering condition was kept constant throughout the present study, with the same Intralipid concentration used for all PpIX-concentration and fluence-rate measurements, the basis spectra did not need to be re-derived for each experimental condition. The basis spectral shapes remained stable across the investigated PpIX concentrations, while changes in the relative contribution of each component were captured by the corresponding SVD fitting coefficients. For MSOLD, SNR was calculated for each spectrum as the fitted peak amplitude divided by the standard deviation of the spectral fitting residual, defined as the measured spectrum minus the fitted spectrum. Across the investigated PpIX concentrations and fluence rates, the MSOLD SNR ranged from 15.56 to 44.61. Spectral decomposition yielded *R*^2^ values of 0.9962–0.9999, indicating excellent fit quality and minimal spectral crosstalk. Although additional measurement uncertainty may arise from excitation-light fluctuations, detector noise, and variability in phantom preparation, the error bars reported for the MSOLD results primarily reflect the uncertainty associated with SVD fitting and the variability of the extracted ^1^O_2_ signal among multiple spectral acquisitions within a single MSOLD measurement under each experimental condition.

Figure 1b shows a representative example of this decomposition, where the measured spectrum is accurately reconstructed by fitting contributions from direct light, PpIX luminescence, and ^1^O_2_ emission. The ^1^O_2_ component extracted from this decomposition provides the instantaneous ^1^O_2_ signal at each time point. Cumulative singlet oxygen production was subsequently calculated by integrating the instantaneous signal over the irradiation period.

Because MSOLD provides continuous spectral acquisition, ^1^O_2_ dynamics can be monitored in real-time throughout irradiation. For comparison with TSOLD measurements, instantaneous signals extracted at the beginning and end of the acquisition period were used to quantify irradiation-induced changes. In addition, comparison of cumulative signals derived from SOED calculations and MSOLD measurements provides a model-based assessment of their proportional relationship under the investigated conditions.

### 2.4. Supporting SOED Calculation for Comparison with MSOLD

Singlet Oxygen Explicit Dosimetry (SOED) calculations were performed as a model-based comparison with cumulative MSOLD measurements. The calculations were based on the established empirical macroscopic singlet-oxygen model derived from Type II PDT photochemistry [23,27]. In this framework, the short-lived excited photosensitizer and singlet-oxygen states are represented through steady-state approximations, yielding reduced expressions describing photosensitizer consumption and cumulative reacted ^1^O_2_. Experimentally measured photosensitizer concentration, dissolved oxygen concentration and delivered light fluence rate were used as model inputs.

The macroscopic SOED expressions used in the present analysis follow the established formulation described in Refs. [23,27]. The corresponding expressions for photosensitizer consumption, oxygen dependence, and cumulative reacted ^1^O_2_ are given below.

(1) dS0dt=−ξO23O23+βϕS0σS0+δ,

(2) dO23dt=−ξϕS0O23+βO23,

(3) O23rx=∫ξO23O23+βϕS01σS0+δ+1dt,

Here [*S*_0_] is the photosensitizer concentration, O23 is the dissolved (ground-state) molecular oxygen concentration, ϕ denotes the fluence rate. The remaining parameters for SOED calculation are listed in Table 1. For the present sealed liquid phantom, the macroscopic SOED model was simplified to reflect experimental conditions. The oxygen-replenishment term $g(1-\frac{\left[O_{2}\right]}{{\left[O_{2}\right]}_{0}})$, which represents external oxygen replenishment in tissue-based implementations of the model, was set to zero because the phantom had no vascular perfusion or imposed external oxygen source. In addition, because no appreciable pre- to post-irradiation change in dissolved oxygen concentration was observed, O23 was treated as constant in the present SOED calculations. This assumption does not exclude transient oxygen changes during irradiation, which were not temporally resolved by the endpoint oxygen measurements.

SOED calculations were used solely for model-based comparison with cumulative ^1^O_2_ signals measured by MSOLD and were not part of the primary comparison between MSOLD and TSOLD. The parameter β characterizes the oxygen dependence of photosensitizer triplet-state quenching in the macroscopic SOED model. Practically, it defines the oxygen concentration scale below which singlet-oxygen production becomes increasingly limited by oxygen availability. The parameter Δ is an empirical low-photosensitizer-concentration correction used in the macroscopic SOED formulation to account for concentration-dependent photobleaching behavior, particularly at low photosensitizer concentrations.

At room temperature (approximately 22 °C), the phantom oxygen partial pressure measured using an OxyLite™ system (Oxford Optronix Ltd., Adderbury, UK) was approximately 140 mmHg, corresponding to an estimated dissolved oxygen concentration of approximately 181 μM based on a conversion factor of 1.29 μM/mmHg. No appreciable pre- to post-irradiation change was observed, supporting the use of constant oxygen concentration as a simplifying assumption for the present model-based calculation. The extinction coefficient of PpIX in methanol was independently determined at 628 nm from the linear relationship between absorbance and PpIX concentration. The measurements were performed in non-scattering methanol solutions without Intralipid. The measured decadic extinction coefficient was approximately 4.6 × 10^3^ M^−1^ cm^−1^, corresponding to approximately 1.06 × 10^4^ M^−1^cm^−1^ after conversion to the Napierian form used in the attenuation calculation. Because the extinction-coefficient measurements were performed without Intralipid, no scattering correction was required for determination of the extinction coefficient itself. Absorbance remained linear over the investigated concentration range, consistent with Beer–Lambert behavior and suggesting that substantial concentration-dependent PpIX aggregation did not affect the absorption at the irradiation wavelength under the experimental conditions used here.

### 2.5. Time-Resolved Singlet Oxygen Luminescence Detection (TSOLD)

**Figure 2:**
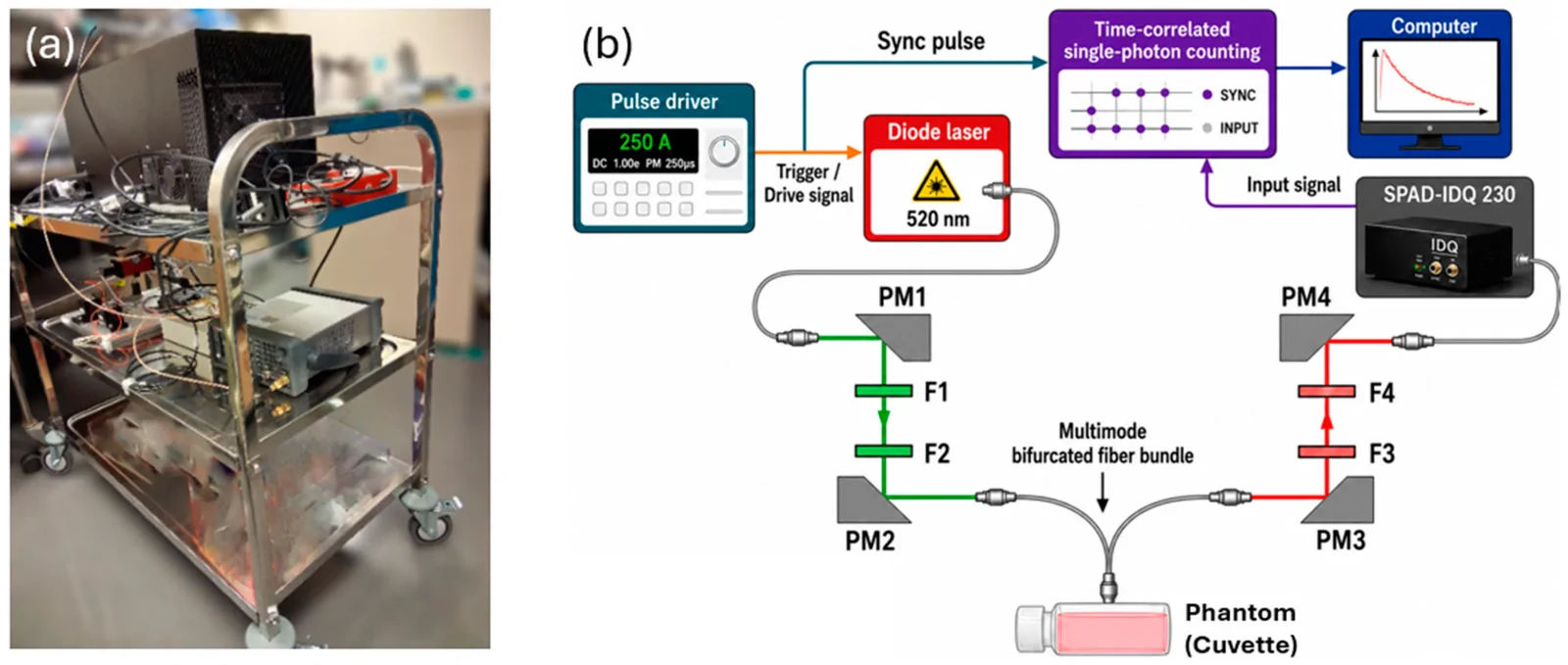
Semi-Transportable TSOLD setup for real-time singlet oxygen dosimetry. (**a**) The time-resolved singlet oxygen luminescence detection (TSOLD) system consists of a nanosecond pulse diode laser, pulse driver, single-photon avalanche diode (SPAD), time-correlated single-photon counting (TCSPC) module, and a bifurcated fiber bundle to illuminate the phantom in a cuvette [16]. (**b**) Schematic of the TSOLD measurement setup for liquid phantom. PM1-PM4 are parabolic mirrors; F1, short-pass filter (FESH0950, 950 nm cut-off); F2, band-pass filter (FGS550, ~304–785 nm); F3, long-pass filter (FELH1200, 1200 nm cut-on); and F4, narrow band-pass filter (1270BP20, 1270 ± 10 nm). Green arrow indicates the excitation-light path; red arrow indicates the detected luminescence path; the orange arrow indicates the trigger/drive signal from pulse driver to diode laser; blue arrows indicate the synchronization pulse from the pulse driver to the TCSPC module and the output signal from TCSPC module to computer; and the purple arrow indicates the SPAD input signal to the TCSPC module.

Time-resolved singlet oxygen luminescence measurements were performed using an in-house developed bifurcated fiber-based TSOLD system, as shown in Figure 2a. Sample excitation was achieved using a 520 nm pulsed diode laser (NPL52C, Thorlabs, Newton, NJ, USA) modulated by a function generator operating with a repetition rate of 35 kHz, a pulse width of 130 ns, and an optical irradiance of 5 mW/cm^2^. The 520 nm excitation wavelength was selected to maximize PpIX absorption efficiency under pulsed excitation while remaining compatible with SPAD based single photon detection. The instrument response function (IRF) of the SPAD detector was approximately 230 ps. The peak irradiance of the 520 nm excitation was estimated from the average irradiance (5 mW/cm^2^), pulse width (130 ns), and repetition rate (35 kHz) to be approximately 1.1 W/cm^2^. Over the 100 s TSOLD acquisition window, this corresponded to approximately 3.5 × 10^6^ excitation pulses. As illustrated in Figure 2b, the excitation beam was directed using a parabolic mirror (RC04FC-P01, Thorlabs) and spectrally conditioned using a short-pass filter (FESH0950, Thorlabs; OD 5 above 950 nm) together with a band-pass filter (FGS550, Thorlabs) to suppress unwanted spectral components. Excitation light was delivered to the sample in a cuvette through a bifurcated fiber bundle (FCR-7UVIR200-2-ME-FC, Avantes, Lafayette, CO, USA) comprising fibers with a 200 μm core diameter and a numerical aperture of 0.22. A bin width of 50 ns was used for the TCSPC histograms, and the SPAD detector dead time was 29 μs. To minimize TCSPC pile-up effects, the detected photon count rate was maintained below approximately 5% of the laser synchronization rate.

Following excitation, singlet-oxygen luminescence was collected through the same bifurcated fiber bundle and directed to the detection system, which included a multimode in-line fiber filter mount (FOFMA/M, Thorlabs), a long-pass filter with a 1200 nm cut-on wavelength (FELH1200, Thorlabs), and a narrow band-pass filter centered at 1270 nm with a 20 nm bandwidth (1270BP20, Omega Optical, Brattleboro, VT, USA) to isolate the ^1^O_2_ emission. The filtered signal was detected using a single-photon avalanche diode (SPAD) detector (ID230, ID Quantique, Geneva, Switzerland). The signal-to-noise ratio (SNR) was calculated as the ratio of the mean ^1^O_2_ signal intensity to the standard deviation of the background noise. The signal was determined from the measured ^1^O_2_ luminescence region, whereas the noise was estimated from a corresponding background region containing no detectable ^1^O_2_ signal.

Photon arrival times were recorded using a time-correlated single photon counting (TCSPC) module (Time Tagger Ultra, Swabian Instruments, Stuttgart, Germany), receiving START signals from the function generator and STOP signals from the SPAD detector output. This approach enables separation of singlet oxygen phosphorescence from short-lived background signals, improving detection specificity under low signal-to-noise conditions. Instrument response functions were measured prior to each experiment, and background signals acquired from methanol phantoms without PpIX were subtracted from all measurements. Temporal luminescence decay profiles were fitted using exponential decay models to extract ^1^O_2_ and photosensitizer triplet-state lifetimes, consistent with established SOLD analysis methods [3,4,5,15,19].

System parameters such as acquisition time, detector dead time, and temporal bin width were optimized to ensure reliable lifetime estimation. Time-resolved photon histograms were analyzed using bi-exponential curve fitting to separate ^1^O_2_ phosphorescence from photosensitizer triplet-state dynamics. The ^1^O_2_ concentration evolution following pulsed excitation is described by [3,4,5,13]

(4) [O21](t)=AτDτT−τDe−t/τTe−t/τD,

where [^1^*O*_2_](*t*) represents ^1^O_2_ concentration as a function of time, *A* = *Nσ*[*S*_0_]*ϕ_D_*, N is the photon fluence per pulse, σ is the photosensitizer absorption cross-section, [*S*_0_] is the photosensitizer concentration, and *ϕ_D_* is the ^1^O_2_ quantum yield. The parameters *τ_D_* and *τ_T_* correspond to the ^1^O_2_ lifetime and photosensitizer triplet lifetime, respectively. Following background subtraction, the time-resolved photon histograms were analyzed using bi-exponential curve fitting based on Equation (4) to separate the ^1^O_2_ phosphorescence component from the photosensitizer triplet-state contribution and other short-lived background components [3,13]. The fitting provided the amplitude *A*, ^1^O_2_ lifetime *τ_D_*, and photosensitizer triplet-state lifetime *τ_T_*. The uncertainties reported for the fitted parameters represent standard errors obtained from the bi-exponential fitting procedure. Fit quality was evaluated using the coefficient of determination (*R*^2^), and the signal-to-noise ratio (SNR) was used to characterize the detected ^1^O_2_ signal. TSOLD measurements were performed immediately before and after irradiation under identical acquisition and detection settings. For the present comparison, the TSOLD endpoint was defined from the fitted pre- and post-irradiation amplitudes after subtraction of the corresponding background amplitude *A*_0_, such that *A_pre_*_, *corr*_ = *A_pre_* − *A*_0_ and *A_post_*_, *corr*_ = *A_post_* − *A*_0_. The irradiation-induced percentage reduction was then calculated as $\%{∆}_{\mathrm{TSOLD}}=\frac{A_{\mathrm{pre},\;\mathrm{corr}}-A_{\mathrm{post},\;\mathrm{corr}}}{A_{\mathrm{pre},\;\mathrm{corr}}}\times 100\%$. Because TSOLD measurements were acquired only before and after irradiation, these values represent fitted pre- and post-irradiation measurements rather than instantaneous or continuous monitoring of ^1^O_2_ production during the 900 s irradiation.

## 3. Results

### 3.1. Singlet Oxygen Signal Dependence on Photosensitizer Concentration

**Figure 3:**
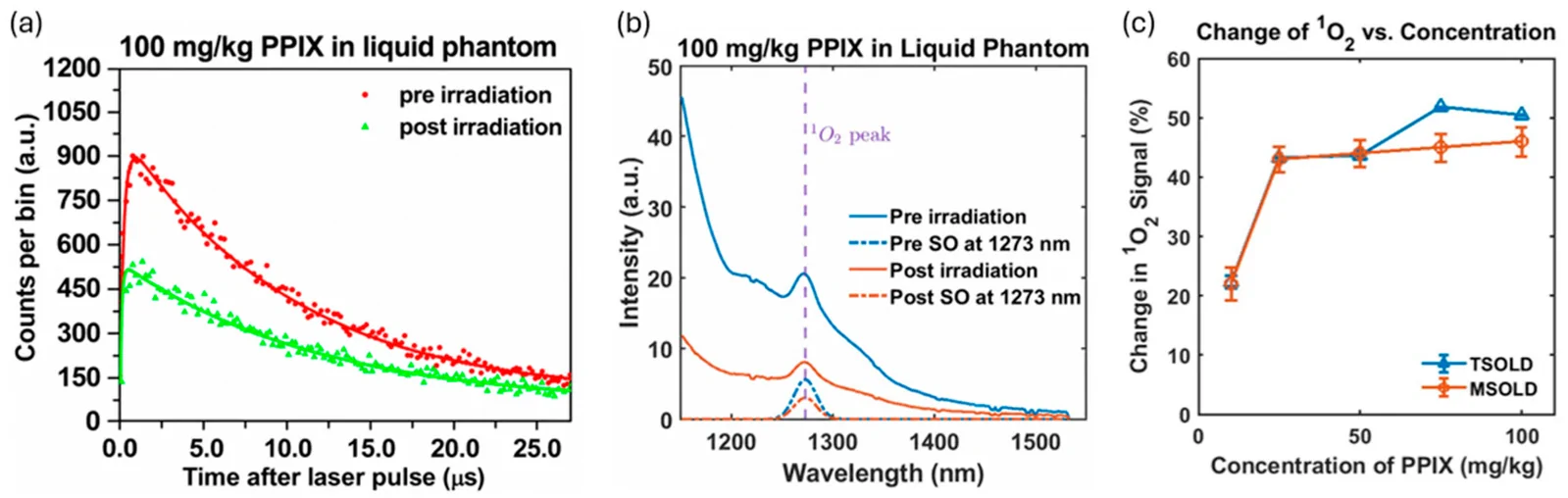
Comparison of TSOLD and MSOLD measurements at different PpIX concentrations. Time-resolved singlet oxygen luminescence detection (TSOLD) and multispectral singlet oxygen luminescence dosimetry (MSOLD) measurements were compared before and after irradiation in liquid protoporphyrin IX (PpIX) phantoms. (**a**) Time-resolved photon counting histograms at 1270 nm for phantoms containing 100 mg/kg PpIX with symbols indicating measured data and solid lines representing fitted decay curves. (**b**) MSOLD emission spectra acquired before and after irradiation at matched photosensitizer concentrations. The extracted ^1^O_2_ signal component is shown as dashed lines. (**c**) Percentage reduction in the background-corrected fitted TSOLD amplitude between the pre- and post-irradiation measurements and in the MSOLD ^1^O_2_ signal between the beginning and end of irradiation, as a function of PpIX concentration.

Figure 3 compares ^1^O_2_ luminescence signals measured using TSOLD and MSOLD in liquid phantoms containing different PpIX concentrations before and after irradiation. Representative TSOLD and MSOLD measurements at 100 mg/kg PpIX are shown in Figure 3a and Figure 3b, respectively, while the percentage reductions in ^1^O_2_ signal across all concentrations are summarized in Figure 3c.

Figure 3a shows representative time-resolved photon-counting histograms acquired at 1270 nm before and after irradiation for the phantom containing 100 mg/kg PpIX. The symbols represent the measured photon counts, and the solid lines represent the fitted decay curves. For the present comparison, the fitted ^1^O_2_-associated amplitudes obtained from the pre- and post-irradiation TSOLD histograms were used as discrete endpoint signals after subtraction of the corresponding background amplitude, as described in Section 2.5. Following 630 nm irradiation at 5 W/cm^2^ for 15 min, the fitted TSOLD endpoint signal decreased. As summarized in Figure 3c, the TSOLD percentage reductions were approximately 22%, 43%, 44%, 52%, and 51% for PpIX concentrations of 10, 25, 50, 75 and 100 mg/kg, respectively.

Figure 3b shows representative MSOLD emission spectra acquired before and after irradiation under the same concentration condition of 100 mg/kg PpIX. The extracted ^1^O_2_ spectral component is shown as dashed lines. Unlike TSOLD, which measures time-resolved photon counts during a defined acquisition window, MSOLD enables spectral extraction of the ^1^O_2_ component during continuous irradiation. For quantitative comparison, the pre-irradiation spectrum was obtained by averaging the first five spectral acquisitions, whereas the post-irradiation spectrum was obtained by averaging the last five spectral acquisitions from the same continuous measurement sequence. Because each average represented only a short acquisition interval relative to the total irradiation duration, these averaged values were treated as instantaneous ^1^O_2_ signals at the beginning and end of irradiation and were referred to as the pre- and post-irradiation MSOLD signals, respectively, for comparison with the corresponding TSOLD measurement. Averaging reduced the influence of short-term fluctuations and uncertainty in data collection. Across the concentration-dependent measurements, the MSOLD SNR ranged from 20.85 to 44.61. After irradiation, the extracted ^1^O_2_ component decreased, consistent with reduced ^1^O_2_ production after irradiation. As summarized in Figure 3c, the MSOLD percentage reductions were approximately 22%, 43%, 44%, 45%, and 46% for PpIX concentrations of 10, 25, 50, 75 and 100 mg/kg, respectively.

TSOLD and MSOLD showed generally consistent concentration-dependent changes in ^1^O_2_ luminescence, as shown in Figure 3c. At lower PpIX concentrations, particularly 10 and 25 mg/kg, the percentage reductions measured by the two methods were very similar, indicating that both approaches captured the same irradiation-induced decrease in ^1^O_2_ production under these conditions. This agreement suggests that percentage-change analysis provides a useful basis for comparing TSOLD and MSOLD despite differences in excitation wavelength, detection geometry, and signal acquisition strategy.

At higher PpIX concentrations, some differences between the mean TSOLD and MSOLD percentage reductions were observed. These differences may arise from the distinct measurement strategies of the two techniques, including differences in excitation wavelength, acquisition timing, signal extraction, and sampled detection region. In particular, TSOLD and MSOLD may not probe exactly the same location or collection volume within the liquid phantom. Because local fluence rate, photosensitizer excitation, oxygen availability, photobleaching, and optical attenuation can vary spatially, small differences in measurement geometry can affect the measured ^1^O_2_ response. To estimate the overall experimental variability of TSOLD, the pre-irradiation fitted amplitudes from the five fluence-rate experiments were compared (see Table S2, A_pre_ values). Because all five measurements were performed using phantoms with the same initial PpIX concentration, their pre-irradiation TSOLD signals were expected to be comparable. The five measured amplitudes had a mean of 814.4 and a sample standard deviation of 79.6, corresponding to a coefficient of variation (CV = SD/mean × 100%) of 9.77%. This variability reflects combined contributions from phantom preparation, measurement positioning and collection geometry, and TSOLD detection variability, rather than geometry alone. Therefore, the observed differences between TSOLD and MSOLD should be interpreted in the context of this overall experimental variability and the differing measurement characteristics of the two systems.

In addition to providing fitted pre- and post-irradiation ^1^O_2_-associated amplitudes, TSOLD also provides lifetime information through time-resolved decay analysis. The decay fitting parameters extracted from TSOLD measurements are summarized in Table 2. The uncertainties reported in the table represent standard errors derived from the nonlinear decay fitting and therefore quantify the uncertainty associated with estimating each fitted parameter from an individual measurement. Because only one measurement was performed for each experimental condition, these values should not be interpreted as sample-to-sample standard deviations or as measures of experimental repeatability.

Across all concentrations, ^1^O_2_ lifetimes remained stable at approximately 10 μs, without systematic dependence on concentration. The PpIX triplet state lifetime does not show observable change (0.26–0.34 μs), considering the measurement uncertainty of the triplet lifetime is 0.1 μs. This is indicative of constant oxygen concentration within the liquid phantom. Fitting quality remained high, with R^2^ values ranging from 0.96 to 0.99. Signal-to-noise ratios increased with concentration, from approximately 10.7 at 10 mg/kg to nearly 19.5 at higher concentrations, explaining the reduced variability observed in TSOLD measurements at higher photon yields. The lifetimes of singlet oxygen and the photosensitizer triplet state can be influenced by the local solvent environment, temperature, oxygen concentration, and the presence of quenchers. The inverse of singlet-oxygen lifetime should be linearly proportional to PpIX concentration. At higher PpIX concentrations, aggregation, self-quenching, and triplet–triplet interactions may also slightly alter the measured lifetimes. Because the experimental conditions were otherwise maintained consistently, the small concentration dependence observed in the singlet oxygen lifetime likely reflects weak concentration-dependent photophysical effects, together with minor uncertainty arising from the signal-to-noise ratio and lifetime fitting.

Overall, both TSOLD and MSOLD captured irradiation-induced reductions in ^1^O_2_ luminescence across the investigated PpIX concentration range. MSOLD provides spectral extraction and continuous monitoring of relative ^1^O_2_ signal changes during irradiation, making it suitable for dynamic monitoring during irradiation. TSOLD, although more limited by photon statistics at low concentrations, provides highly specific lifetime-resolved detection and additional mechanistic information that cannot be directly obtained from MSOLD. Together, the agreement between the two methods supports their complementary use for characterizing and monitoring irradiation-induced ^1^O_2_ dynamics.

### 3.2. Comparison of MSOLD and TSOLD Under Varying Fluence Rates

To further compare MSOLD and TSOLD performance under different irradiation conditions, measurements were conducted using a fixed PpIX concentration (50 mg/kg) while varying the irradiation fluence rate from 1 to 5 W/cm^2^. In this study, the terms fluence rate and irradiance are used interchangeably to describe the incident optical power per unit area.

**Figure 4:**
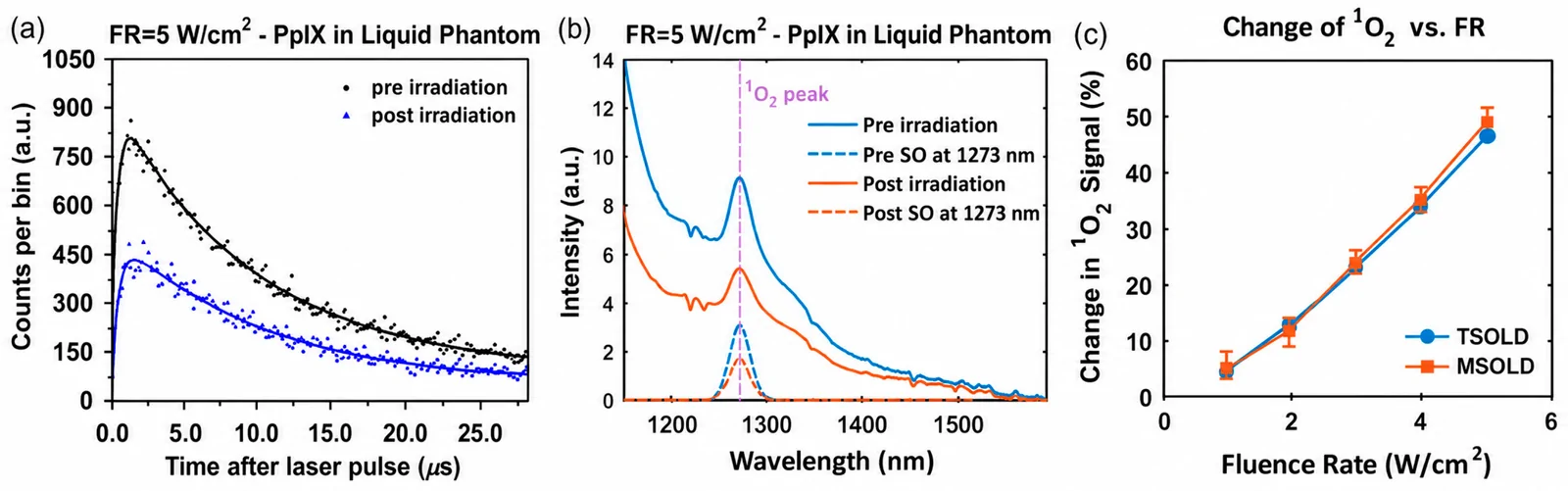
Comparison of TSOLD and MSOLD measurements at different fluence rates. Time-resolved singlet oxygen luminescence detection (TSOLD) and multispectral singlet oxygen luminescence dosimetry (MSOLD) measurements were compared in liquid phantoms containing 50 mg/kg (88.9 µmol/kg) protoporphyrin IX (PpIX). (**a**) TSOLD photon counting histograms measured before and after irradiation at a representative fluence rate of 5 W/cm^2^. (**b**) MSOLD emission spectra before and after irradiation under the same conditions. The extracted ^1^O_2_ signal component is shown as dashed lines. (**c**) Percentage reduction in the background-corrected fitted TSOLD amplitude between the pre- and post-irradiation measurements and in the MSOLD ^1^O_2_ signal between the beginning and end of irradiation, as a function of fluence rate.

Figure 4a shows representative TSOLD time-resolved photon-counting histograms acquired before and after irradiation at a fluence rate of 5 W/cm^2^. The fitted decay curves demonstrate a clear reduction in ^1^O_2_ luminescence after irradiation. The reduction became more pronounced with increasing fluence rate, demonstrating a stronger irradiation-dependent change in the measured ^1^O_2_ luminescence signal. The percentage decreases in cumulative TSOLD signals were approximately 4%, 15%, 26%, 34%, and 46% at fluence rates of 1, 2, 3, 4, and 5 W/cm^2^, respectively, as summarized in Figure 4c.

Representative MSOLD measurements acquired under the same conditions are shown in Figure 4b. The pre- and post-irradiation emission spectra show a clear decrease in the extracted ^1^O_2_ signal component at 1273 nm after irradiation. The extracted ^1^O_2_ signal components are shown as dashed lines. Across the fluence-rate-dependent measurements, the MSOLD SNR ranged from 15.56 to 23.80. Similar to TSOLD, the MSOLD percentage reduction increased with fluence rate, with changes of approximately 5%, 11%, 24%, 35%, and 48% at fluence rates of 1, 2, 3, 4, and 5 W/cm^2^, respectively. As shown in Figure 4c, TSOLD and MSOLD exhibited the same fluence-rate-dependent trend. The two methods showed small differences at all fluence rates.

The progressively larger ^1^O_2_ signal reduction with increasing fluence rate indicates a stronger fluence-rate-dependent effect on the measured ^1^O_2_ luminescence. Higher irradiance increases photosensitizer excitation and may alter multiple photochemical and physical processes in the phantom, including photosensitizer photobleaching, transient changes in oxygen availability, and temperature-dependent effects. Consistent with these fluence-rate-dependent effects, larger reductions in ^1^O_2_ luminescence were observed at higher irradiances. The fitted TSOLD pre- to post-irradiation signal changes and the corresponding MSOLD changes from the beginning to the end of irradiation showed a similar irradiance-dependent trend. The general consistency between the two methods across different fluence rates indicates that both detected fluence-rate-dependent changes in ^1^O_2_ luminescence. Because TSOLD and MSOLD use different excitation wavelengths, detection geometries, and acquisition schemes, their absolute signal magnitudes are not directly comparable. Therefore, the comparison in Figure 4c was based on percentage reduction rather than absolute signal intensity, which reduces the influence of system-specific signal scaling. The similar trends observed with increasing fluence rate support that both techniques are sensitive to irradiation-dependent changes in ^1^O_2_ luminescence under the experimental conditions studied.

The corresponding ^1^O_2_ and PpIX triplet-state lifetimes derived from TSOLD decay fitting, together with fitting quality (R^2^) and signal-to-noise ratio (SNR), are summarized in Table 3. The reported uncertainties are fit-derived standard errors and do not represent sample-to-sample variability. Across all fluence rates, ^1^O_2_ lifetimes remained stable near 10.5 μs, indicating that oxygen relaxation dynamics in the liquid phantom were largely unaffected by irradiation intensity. Similarly, PpIX triplet lifetimes remained within a narrow range of approximately 0.28–0.32 μs, showing no systematic dependence on fluence rate. Fitting quality remained high with R^2^ values exceeding 0.98, confirming robust decay fitting. SNR values ranged from approximately 17 to 20, demonstrating stable photon statistics across fluence conditions.

Together, the fluence-rate-dependent results demonstrate that both TSOLD and MSOLD capture progressively larger irradiation-induced reductions in ^1^O_2_ luminescence with increasing irradiance. The similar trends observed with the two techniques indicate that both are sensitive to fluence-rate-dependent changes in the measured ^1^O_2_ luminescence, despite differences in excitation wavelength, detection geometry, and signal acquisition strategy. By comparing percentage changes rather than absolute signal intensity, these instrumental differences are largely normalized, leading to the similar trends observed in Figure 4c. Overall, TSOLD provides lifetime-resolved and cumulative ^1^O_2_ measurements that are valuable for mechanistic validation, whereas MSOLD enables real-time, continuous spectral monitoring that is well suited for real-time PDT dosimetry.

### 3.3. Comparison of MSOLD Measurements with SOED Calculations

To examine the relationship between the measured MSOLD signal and model-calculated reacted ^1^O_2_, cumulative MSOLD-derived ^1^O_2_ signals were compared with SOED calculations under matched experimental conditions. This analysis provides a model-based comparison between two methodologically distinct estimates of ^1^O_2_-related quantities. MSOLD derives the ^1^O_2_ signal directly from spectral decomposition of the measured luminescence spectra, whereas SOED calculates reacted ^1^O_2_ from the measured photosensitizer concentration, dissolved oxygen concentration, delivered light fluence rate, and established photophysical parameters.

**Figure 5:**
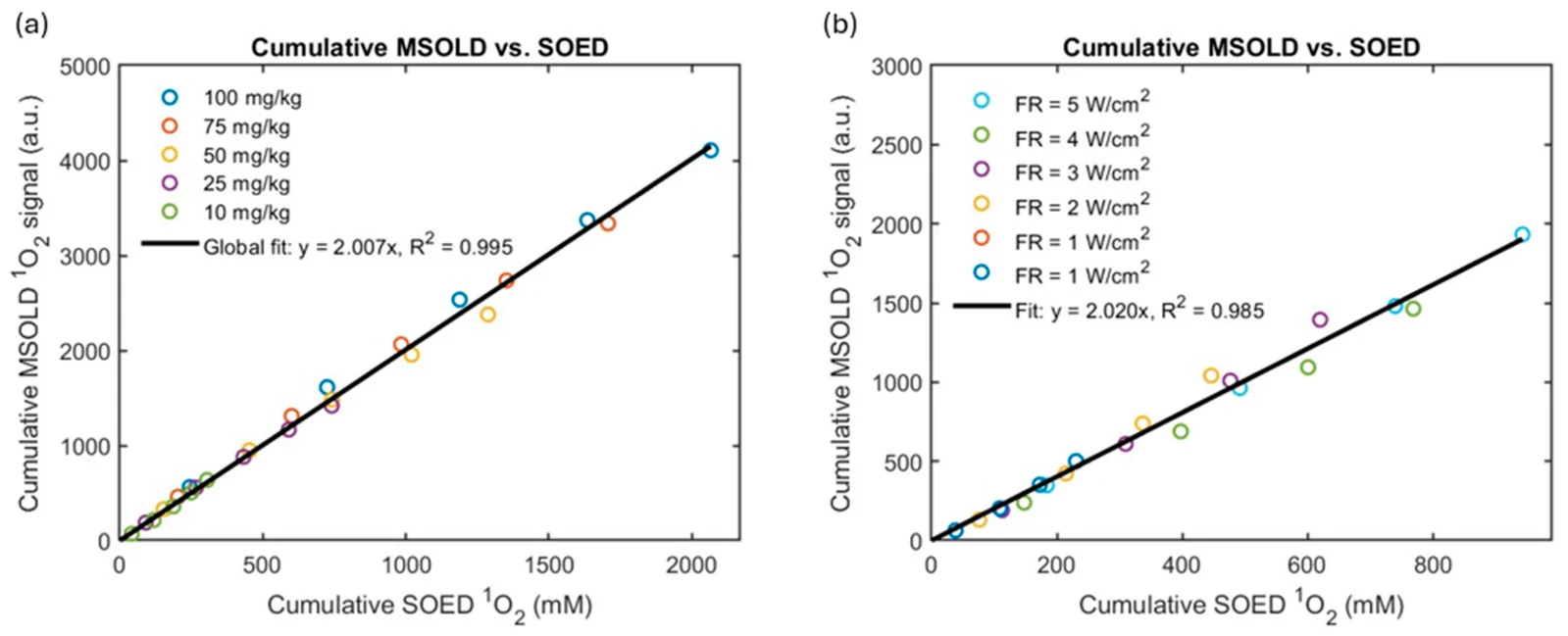
Relationship between cumulative MSOLD-derived and SOED-calculated ^1^O_2_. Cumulative multispectral singlet oxygen luminescence dosimetry (MSOLD)-derived ^1^O_2_ signals were compared with singlet oxygen explicit dosimetry (SOED)-calculated reacted ^1^O_2_. (**a**) Data acquired at different protoporphyrin IX (PpIX) concentrations under a fixed fluence rate. (**b**) Data acquired at different fluence rates under a fixed PpIX concentration. Different markers indicate different concentrations or fluence rates. Solid lines represent global linear fits to all data points in each panel.

Figure 5 shows the relationship between the cumulative MSOLD-derived ^1^O_2_ signal and SOED-calculated reacted ^1^O_2_ under two experimental settings. Figure 5a shows data acquired at different PpIX concentrations under a fixed fluence rate, while Figure 5b shows data acquired at different fluence rates under a fixed PpIX concentration. Because both cumulative quantities are zero at the start of irradiation, data from all experimental conditions and selected time points in each panel were fitted using a single global linear regression constrained through the origin. The concentration-dependent dataset yielded a slope of 2.007 with an R^2^ value of 0.995, while the fluence-rate-dependent dataset yielded a similar slope of 2.020 with an R^2^ value of 0.985. The absolute difference between the two slopes was only 0.013, corresponding to a relative difference of approximately 0.65% when normalized to their average value. The close agreement between the fitted slopes indicates a similar proportional relationship between the cumulative MSOLD signal and SOED-calculated reacted ^1^O_2_ across the two experimental conditions.

These strong linear correlations and similar slopes indicate that MSOLD- and SOED-predicted cumulative ^1^O_2_ production tracked similarly under the conditions examined across variations in both photosensitizer concentration and fluence rate. The similarity between the two datasets suggests that the proportional relationship between MSOLD-derived signal and SOED-calculated reacted ^1^O_2_ is preserved across the concentration- and fluence-rate-dependent experimental conditions.

The slope of the MSOLD–SOED relationship provides an empirical proportionality factor under the present experimental conditions. Because similar slopes were obtained for the concentration- and fluence-rate-dependent datasets, the proportional relationship appeared similar across the two experimental series. However, because the cumulative time points are correlated and no independently replicated experimental series were performed, this proportionality factor should not be interpreted as a validated calibration coefficient. Independent replicate studies will be required to determine whether such a relationship can support quantitative calibration of MSOLD.

In summary, Figure 5 demonstrates an approximately linear model-based relationship between cumulative MSOLD signal and SOED-calculated reacted ^1^O_2_ under the investigated conditions. The observed proportional relationship between MSOLD and SOED across both concentration- and fluence-rate-dependent experiments supports the use of MSOLD as a real-time luminescence-based dosimetry method for monitoring irradiation-induced ^1^O_2_ dynamics. Together with the TSOLD results described in Section 3.1 and Section 3.2, these findings suggest that MSOLD offers a practical approach for continuous PDT dosimetry, while SOED provides a complementary model-based framework for comparison.

## 4. Discussion

Based on one measurement with each system for every experimental condition, TSOLD and MSOLD exhibited similar trends in irradiation-induced changes in ^1^O_2_ luminescence across the investigated conditions. TSOLD measurements were acquired before and after irradiation using identical excitation and detection settings, with lifetime-resolved ^1^O_2_ photon counts accumulated following pulsed 520 nm excitation over a defined acquisition window. The pre-irradiation TSOLD signal was therefore determined primarily by the initial PpIX concentration and oxygen availability, whereas the post-irradiation signal additionally reflected irradiation-induced dynamic changes, including photosensitizer photobleaching and changes in oxygen concentration [30]. In contrast, MSOLD measurements were acquired continuously during 630 nm irradiation and thus directly tracked changes in the spectrally resolved ^1^O_2_ signal throughout irradiation. Consequently, the relative change between the pre- and post-irradiation TSOLD measurements provides an endpoint measure of the overall irradiation-induced change and can be compared with the real-time changes observed using MSOLD during irradiation.

In the concentration-dependent measurements, TSOLD and MSOLD showed generally consistent behavior, particularly at lower PpIX concentrations, where the percentage reductions measured by the two methods were closely aligned. This agreement suggests that percentage-change analysis provides a useful basis for comparing TSOLD and MSOLD when the photochemical response is not strongly affected by oxygen depletion, optical attenuation, or nonlinear photosensitizer behavior. At higher PpIX concentrations, some differences between the mean TSOLD and MSOLD percentage reductions were observed. These differences may arise from small variations in measurement geometry, since TSOLD and MSOLD may not sample exactly the same location or collection volume within the liquid phantom. Local fluence rate, photosensitizer excitation, oxygen availability, photobleaching, and optical attenuation can vary spatially, and this effect becomes more pronounced at higher photosensitizer concentrations where self-shielding and photobleaching are stronger.

Differences in excitation wavelength may also contribute to residual differences between the two methods. TSOLD uses pulsed excitation at 520 nm before and after the 630 nm irradiation period, whereas MSOLD measures ^1^O_2_ luminescence during continuous 630 nm irradiation. Because PpIX absorption, tissue or phantom light propagation, and excitation efficiency are wavelength-dependent, the two excitation schemes may produce different absolute signal amplitudes, triplet-state populations, oxygen-consumption rates, and photobleaching behavior. However, the present comparison is based primarily on the percentage change in the ^1^O_2_ signal measured independently by each system rather than on a direct comparison of absolute signal intensity. Previous Monte Carlo modeling has shown that wavelength-dependent differences in reacted singlet-oxygen concentration can be represented largely by a multiplicative correction factor under otherwise comparable conditions [31]. If this wavelength-dependent scaling factor remains approximately constant over the tested conditions, its influence is largely canceled when each signal is normalized to its corresponding baseline (pre-irradiation) value. Therefore, although the difference in excitation wavelength remains a limitation, it is not expected to substantially affect the comparison of relative irradiation-induced changes measured by TSOLD and MSOLD.

The fluence-rate-dependent measurements showed similar trends between TSOLD and MSOLD, with both methods exhibiting progressively larger percentage reductions in ^1^O_2_ luminescence as fluence rate increased. The stronger signal reduction at higher irradiances likely reflects the combined influence of multiple irradiance-dependent processes rather than a single mechanism. Because photosensitizer photobleaching, transient oxygen dynamics, and thermal effects were not independently resolved in the present study, their relative contributions to the observed changes in ^1^O_2_ luminescence cannot be determined. Moreover, the endpoint dissolved-oxygen measurements showed no appreciable pre- to post-irradiation decrease and therefore do not support attributing the observed signal reduction primarily to oxygen depletion. Nevertheless, the similar fluence-rate-dependent trends observed with TSOLD and MSOLD indicate that both techniques were sensitive to irradiation-dependent changes in ^1^O_2_ luminescence under the experimental conditions investigated.

The comparison between MSOLD and SOED further demonstrates a consistent model-based proportional relationship between cumulative MSOLD signal and SOED-calculated reacted ^1^O_2_ under the investigated experimental conditions. Although the cumulative MSOLD signal is expressed in arbitrary units, its approximately linear relationship with SOED-calculated reacted ^1^O_2_ suggests the potential for establishing an empirical scaling relationship under defined experimental conditions. When the MSOLD–SOED relationship was fitted using a linear regression constrained through the origin, proportionality coefficients of 2.007 and 2.020 were obtained for the concentration-dependent and fluence-rate-dependent datasets, respectively. The similarity of these coefficients suggests a consistent scaling relationship between cumulative MSOLD and SOED across the two investigated datasets. However, because the cumulative time points within each irradiation trajectory are correlated and the comparison was not performed using independent replicate experiments, these regression results should be interpreted as a model-based comparison rather than independent validation or calibration. Further studies using independently replicated measurements will be required to determine whether SOED can serve as a quantitative reference for calibration of the MSOLD signal.

The MSOLD–SOED comparison also provides supporting context for the TSOLD measurements. Because TSOLD and MSOLD showed similar concentration- and fluence-rate-dependent trends, the model-based relationship between MSOLD and SOED is consistent with the interpretation that both optical techniques are sensitive to irradiation-induced changes in ^1^O_2_ luminescence. Together, SOED, MSOLD, and TSOLD provide complementary information: SOED provides a model-based reference, MSOLD enables real-time spectral monitoring consistent with recent high-throughput spectroscopic ^1^O_2_ and photosensitizer luminescence dosimetry approaches, and TSOLD provides lifetime-resolved confirmation of ^1^O_2_ luminescence [32].

From a potential translational perspective, the continuous acquisition capability of MSOLD may ultimately be useful for treatment monitoring and adaptive dosimetry, consistent with the broader development of quantitative and personalized PDT dosimetry strategies [33], although the present phantom study does not establish performance under in vivo or clinical conditions. In contrast, TSOLD provides lifetime-resolved information that is useful for mechanistic studies and characterization of singlet-oxygen kinetics. TSOLD may also potentially be implemented for treatment surveillance when modulated or pulsed excitation is incorporated during irradiation, although this acquisition mode was not evaluated in the present study. MSOLD and TSOLD therefore provide distinct and complementary measurement capabilities. In the present experiment, MSOLD enables continuous spectral monitoring during irradiation, whereas TSOLD provides lifetime-resolved information from measurements acquired before and after irradiation. Because the present study did not include a formal comparison of detection limits, acquisition time, dynamic range, or temporal resolution, no definitive performance advantage between the two platforms can be established.

Several limitations should be acknowledged. A primary limitation of the present study is the absence of independent experimental replicates. One independently prepared phantom was evaluated for each PpIX-concentration and fluence-rate condition. Consequently, the fit-derived standard errors and variability among repeated spectra within an individual acquisition reflect fitting and within-measurement uncertainty but do not characterize sample-to-sample reproducibility. The present results should therefore be interpreted as an exploratory cross-platform comparison demonstrating similarity in the observed concentration- and fluence-rate-dependent trends rather than as a statistical demonstration of equivalence or repeatability between MSOLD and TSOLD. Future studies incorporating independently prepared replicate phantoms will be required to quantify inter-sample variability, repeatability, and cross-platform agreement.

Measurements were performed in homogeneous liquid phantoms, which do not fully represent the optical heterogeneity, vascular oxygen supply, or dynamic biological environment of in vivo tissues. The methanol-rich phantom was selected as a controlled and reproducible solution-based scattering system for comparison of MSOLD and TSOLD rather than as a biologically representative or tissue-mimicking phantom. The phantom remained visually stable during the experimental period, with no apparent phase separation or precipitation. The approximately 10 µs ^1^O_2_ lifetime observed in this system reflects the methanol-dominated environment and is substantially longer than that expected in biological tissue; therefore, this lifetime should be considered phantom-specific and should not be directly extrapolated to in vivo conditions. Additional TSOLD measurements comparing pure methanol with the 0.6% Intralipid scattering phantom showed that the introduction of scattering reduced the fitted signal amplitude by approximately 22%, while the fitted ^1^O_2_ lifetime changed only modestly from 10.51 ± 0.03 μs to 10.05 ± 0.03 μs (Figure S8). This suggests that, under the present experimental conditions, scattering primarily affected the detected signal magnitude rather than substantially altering the extracted ^1^O_2_ lifetime. In particular, the homogeneous phantom environment does not reproduce tissue-specific photosensitizer binding or biological quenching pathways, both of which may alter the magnitude and lifetime of the detected ^1^O_2_ signal in vivo. Future studies should therefore evaluate both detection approaches in more biologically relevant aqueous and protein-containing phantoms with stronger ^1^O_2_ quenching and shorter lifetimes, followed by in vivo evaluation. The relatively high irradiances used here, 1–5 W/cm^2^, were selected deliberately to produce a measurable irradiation-induced reduction in the singlet-oxygen luminescence signal, thereby providing sufficient signal variation for comparison between TSOLD and MSOLD. The irradiances used in the present phantom experiments substantially exceed the approximately 100–300 mW/cm^2^ range commonly reported for conventional PDT light delivery, above which tissue heating may become a concern [34]. Consistent with this, the phantom temperature increased during irradiation by approximately 1.1 °C at 1 W/cm^2^ and 5.8 °C at 5 W/cm^2^. Such temperature changes may influence oxygen solubility, photochemical reaction rates, photosensitizer behavior, and singlet-oxygen lifetime. Accordingly, thermal effects cannot be separated from other irradiance-dependent effects in the present experiment and represent a potential confounding factor when interpreting the fluence-rate-dependent changes in ^1^O_2_ luminescence. Moreover, irradiance is not merely an experimental scaling parameter: under in vivo conditions, it can alter oxygen consumption and replenishment, photosensitizer photobleaching, and the measured singlet-oxygen signal kinetics [35]. Thus, the present high-irradiance phantom results should not be interpreted as directly representative of animal or clinical PDT conditions. Nevertheless, because MSOLD and TSOLD were compared under matched phantom and irradiation conditions, the potential influence of heating does not preclude comparison of the relative fluence-rate-dependent trends observed with the two techniques.

In addition, MSOLD and TSOLD used different excitation wavelengths, detection configurations, and acquisition schemes, which affect absolute emission magnitudes and complicate direct one-to-one comparison. TSOLD performance under low-signal conditions is limited by photon-counting statistics, background subtraction, detector sensitivity, and fitting uncertainty, and may require longer acquisition times to improve signal-to-noise ratio. Nevertheless, the translational relevance of the present phantom-based evaluation is supported by the recent preclinical application of the same TSOLD platform. Using Photofrin-mediated PDT, this system directly detected ^1^O_2_ luminescence from RIF tumors in vivo, where the measured signal was affected by tissue absorption, scattering, heterogeneous photosensitizer distribution, and dynamic oxygen availability [15]. Previous preclinical application of the same TSOLD platform demonstrated the feasibility of detecting ^1^O_2_ luminescence in vivo [15], supporting continued evaluation under biologically relevant conditions. Recent high-sensitivity ^1^O_2_ luminescence sensing strategies using computational spectroscopy and solid-state detection may further improve signal recovery under low-light conditions [36]. Despite these limitations, the controlled phantom framework enabled a systematic comparison of the two techniques. The observed MSOLD-to-SOED proportionality factor (~2.014) should be considered specific to the present experimental conditions, as it may depend on optical properties, photosensitizer environment, measurement geometry, detector response, and excitation wavelength. An integrated approach combining MSOLD, SOED, and TSOLD may provide a framework for further preclinical and translational evaluation of singlet-oxygen-based dosimetry.

## 5. Conclusions

In this exploratory study, MSOLD and TSOLD were systematically compared in a controlled PpIX liquid phantom under matched phantom and irradiation conditions. Both methods showed similar concentration-dependent and fluence-rate-dependent trends in irradiation-induced ^1^O_2_ luminescence changes, supporting their ability to detect irradiation-related ^1^O_2_ dynamics. MSOLD provided continuous monitoring of ^1^O_2_ generation during irradiation, whereas TSOLD provided lifetime-resolved confirmation of ^1^O_2_ luminescence. Importantly, TSOLD also enabled extraction of ^1^O_2_ lifetime and PpIX triplet-state lifetime, providing additional mechanistic information that cannot be directly obtained from steady-state MSOLD measurements. The approximately linear relationship between cumulative MSOLD signal and SOED-calculated reacted ^1^O_2_, with similar proportionality coefficients across the concentration- and fluence-rate-dependent datasets, further demonstrates a model-based proportional relationship under investigated conditions. Taken together, these results support the complementary use of MSOLD and TSOLD for singlet-oxygen-based PDT dosimetry: MSOLD may have potential for real-time treatment monitoring and, following validation in biologically relevant systems, adaptive PDT guidance, while further independently replicated studies are needed to establish reproducibility, quantitative calibration, and broader applicability.

## Figures and Tables

**Table 1:** Photophysical parameters used for SOED calculations. Photophysical parameters for protoporphyrin IX (PpIX) used in the singlet oxygen explicit dosimetry (SOED) calculations. Parameter symbols, values, units, and corresponding references are provided.

Parameter	Definition	In Vitro	In Vivo
*ε* (cm^−1^∙μM^−1^)	Photosensitizer extinction coefficient	0.0106 at 630 nm ^(1)^ 0.0129 at 527 nm ^(2)^	0.003 at 633 nm [27] 0.01 at 523 nm [12]
*β* (μM)	Oxygen-quenching threshold concentration	11.9 [27]	
Δ (μM)	Empirical Low-concentration correction parameter	128 ± 20 [23]	33 [27]
*σ* (μM^−1^)	Specific photobleaching ratio *σ* = *k*_1_*τ*_Δ_	7.41 × 10^−5 (3)^	(9.0 ± 1.6) × 10^−5^ [23]
*τ*_Δ_ (μs)	Singlet oxygen lifetime	10.55 ± 0.23	26 [28]
*τ_t_* (μs)	Triplet state lifetime	0.3 ± 0.02	0.04 [28]
*ξ* (cm^2^mW^−1^s^−1^)	Specific oxygen consumption rate $\xi =\Phi_{\Delta }\frac{\varepsilon }{\mathrm{h\nu}}$	31.3 × 10^−3^ at 630 nm ^(4)^ 31.9 × 10^−3^ at 527 nm ^(5)^	3.7 × 10^−3^ at 633 nm [27] 1.02 × 10^−2^ at 523 nm ^(6)^
Φ_Δ_	Singlet oxygen quantum yield	0.56 [29]	0.233 [27]

(1) *ϵ* was measured with PpIX in methanol phantom at 630 nm; (2) *ϵ* was measured with PpIX in methanol phantom at 527 nm; (3) *σ* was recalculated using *σ* = *k*_1_*τ*_Δ_, where *k*_1_ = 7.02 μM^−1^s^−1^ was estimated from the *σ*_ref_ and *τ*_Δ,ref_ values reported in Ref. [23], giving *σ* = 7.41 × 10^−5^ μM^−1^ using the measured *τ*_Δ_ = 10.55 μs; (4) Calculated using Φ_Δ_ = 0.56, *ε* = 0.0106 cm^−1^∙μM^−1^ and *hν* = 3.14 × 10^−16^ mWs at 630 nm; (5) Calculated using Φ_Δ_ = 0.56, *ε* = 0.0129 cm^−1^∙μM^−1^ and *hν* = 3.77 × 10^−16^ mWs at 527 nm; (6) Calculated using Φ_Δ_ = 0.233, *ε* = 0.01 cm^−1^∙μM^−1^ and *hν* = 3.80 × 10^−16^ mWs at 523 nm.

**Table 2:** TSOLD-derived ^1^O_2_ and PpIX triplet-state lifetimes at different PpIX concentrations. Singlet oxygen (^1^O_2_) and protoporphyrin IX (PpIX) triplet-state lifetimes measured using time-resolved singlet oxygen luminescence detection (TSOLD) in liquid phantoms containing different PpIX concentrations and composed of approximately 97% methanol and 0.6% Intralipid, under an fluence rate of 5 W/cm^2^. Corresponding coefficients of determination (R^2^) and signal-to-noise ratios (SNR) were obtained from bi-exponential fitting of the TSOLD data. Reported uncertainties represent standard errors derived from the nonlinear fitting procedure for individual measurements.

Conc. of PpIX (mg/kg)	Conc. of PpIX (μmol/kg)	Singlet Oxygen Lifetime (µs)	PpIX Triplet State Lifetime (µs)	Coefficient of Determination (R^2^)	Signal-to-Noise Ratio
10	17.8	10.53 ± 0.7	0.26 ± 0.1	0.96	10.7
25	44.4	10.65 ± 0.5	0.30 ± 0.1	0.97	14.8
50	88.9	10.39 ± 0.3	0.28 ± 0.1	0.99	18.7
75	133.3	10.43 ± 0.3	0.34 ± 0.1	0.99	19.5
100	177.73	10.14 ± 0.3	0.29 ± 0.1	0.99	19.5

**Table 3:** TSOLD-derived ^1^O_2_ and PpIX triplet-state lifetimes at different fluence rates. Singlet oxygen (^1^O_2_) and protoporphyrin IX (PpIX) triplet-state lifetimes measured using time-resolved singlet oxygen luminescence detection (TSOLD) at different fluence rates in liquid phantoms containing 50 mg/kg (88.9 µmol/kg) PpIX and composed of approximately 97% methanol and 0.6% Intralipid. Corresponding coefficients of determination (R^2^) and signal-to-noise ratios (SNR) were obtained from bi-exponential fitting of the TSOLD data. Reported uncertainties represent standard errors derived from the nonlinear fitting procedure for individual measurements.

Fluence Rate (W/cm^2^)	Singlet Oxygen Lifetime (µs)	PpIX Triplet State Lifetime (µs)	Coefficient of Determination (R^2^)	Signal-to-Noise Ratio
1	10.88 ± 0.38	0.31 ± 0.04	0.9884	17.98
2	10.58 ± 0.32	0.32 ± 0.04	0.9909	20.16
3	10.91 ± 0.34	0.29 ± 0.04	0.9904	19.62
4	10.59 ± 0.37	0.28 ± 0.04	0.9877	17.49
5	10.41 ± 0.37	0.31 ± 0.04	0.9874	16.93

## Data Availability

The raw spectra, photon-arrival histograms, dissolved-oxygen traces, irradiation records, TSOLD/MSOLD fitting scripts, SVD basis spectra, MSOLD–SOED regression data, and SOED calculation code supporting the findings of this study are available from the corresponding author upon reasonable request.

## Supplementary Materials

The following supporting information can be downloaded at https://www.mdpi.com/article/10.3390/antiox15101250/s1, Figure S1: Raw TSOLD measurements at different PpIX concentrations; Figure S2: Raw MSOLD spectra at different PpIX concentrations; Figure S3: Raw TSOLD measurements at different irradiation fluence rates; Figure S4: Raw MSOLD spectra at different irradiation fluence rates; Figure S5: Comparison of instantaneous and cumulative ^1^O_2_ signals from MSOLD and SOED for different PpIX concentrations; Figure S6: Comparison of instantaneous and cumulative ^1^O_2_ signals from MSOLD and SOED for different fluence rates; Figure S7: Experimental determination of the PpIX extinction coefficient in methanol; Figure S8: Comparison of TSOLD measurements in pure methanol and a methanol–Intralipid scattering phantom; Table S1: Fitted amplitude (A) and background values obtained from histogram fitting at different PpIX concentrations under a constant fluence rate of 5 W/cm^2^; Table S2: Fitted amplitude (A) and background values obtained from histogram fitting at different irradiation fluence rates with a constant PpIX concentration of 50 mg/kg; Table S3: PpIX concentrations measured before and after 900 s of irradiation at different initial PpIX concentrations under a constant fluence rate of 5 W/cm^2^; Table S4: PpIX concentrations measured before and after 900 s of irradiation at different fluence rates under a constant initial PpIX concentration of 50 mg/kg.

## Abbreviations

The following abbreviations are used in this manuscript:

MSOLD	Multispectral Singlet Oxygen Luminescence Dosimetry
PDT	Photodynamic Therapy
PpIX	Protoporphyrin IX
SNR	Signal-to-Noise Ratio
SOED	Singlet Oxygen Explicit Dosimetry
SPAD	Single-Photon Avalanche Diode
TCSPC	Time-Correlated Single-Photon Counting
TSOLD	Time-Resolved Singlet Oxygen Luminescence Dosimetry
