# Supplementary material for: Cross-Platform Comparison of Multispectral and Time-Resolved Singlet Oxygen Luminescence Dosimetry Under Matched Irradiation Conditions in a Liquid Protoporphyrin IX Phantom
Source: Antioxidants (Basel). 2026 Sep 29;15(10):1250. doi: 10.3390/antiox15101250 (PMC13700573; doi:10.3390/antiox15101250)
Supplement: Supplementary file 1 [file antioxidants-15-01250-s001.zip › antioxidants-4412379-supplementary.pdf]

Supplement file

Cross-Platform Comparison of Multispectral and Time-Resolved Singlet Oxygen Luminescence Dosimetry Under Matched Irradiation Conditions in a Liquid Protoporphyrin IX Phantom

Baozhu Lu [1,†], Vikas Vikas [2,†], Weibing Yang [1], Robert H. Hadfield [2], Brian C. Wilson[3] and Timothy C. Zhu [1,*]

[1] Department of Radiation Oncology, University of Pennsylvania, Philadelphia, PA 19104, USA; baozhu.lu@pennmedicine.upenn.edu (B.L.); weibing.yang@pennmedicine.upenn.edu (W.Y.)
[2] James Watt School of Engineering, University of Glasgow, Glasgow G128LT, UK; vikas.vikas.2@glasgow.ac.uk (V.V.); robert.hadfield@glasgow.ac.uk (R.H.H.)
[3] Department of Medical Biophysics, University Health Network, University of Toronto, Toronto, ON M5G 1L7, Canada; brian.wilson@uhn.ca
* Correspondence: timothy.zhu@pennmedicine.upenn.edu
† These authors contributed equally to this work.

1. Complete TSOLD and MSOLD Signals and Fitting for Different PpIX Concentrations at a Constant Fluence Rate and for Different Fluence Rates at a Constant PpIX Concentration (50 mg/kg)

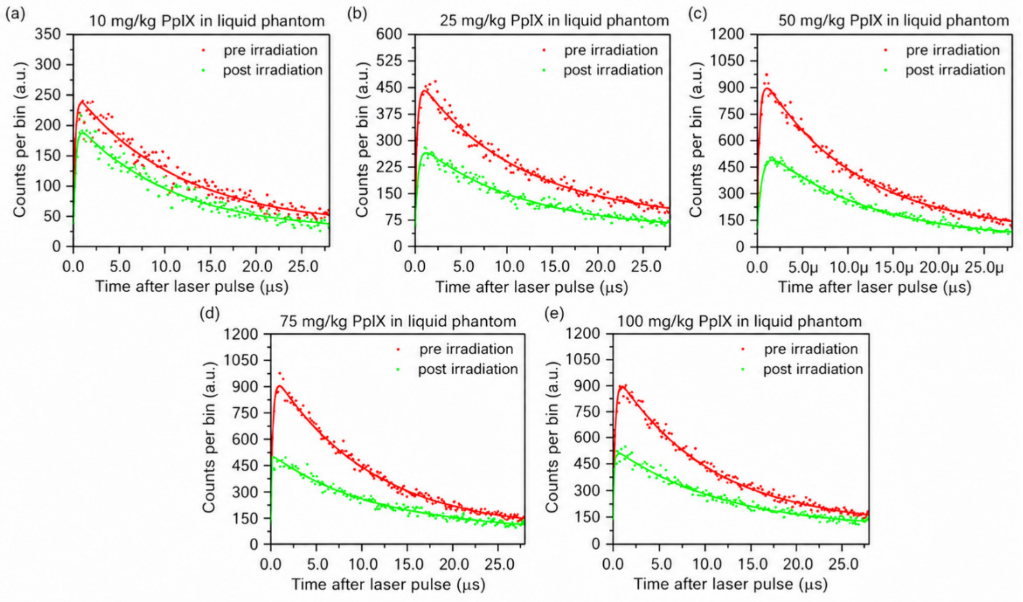

Figure S1. Raw TSOLD measurements at different PpIX concentrations. Time-resolved singlet oxygen luminescence detection (TSOLD) photon-counting histograms acquired before and after irradiation in liquid PpIX phantoms with different photosensitizer concentrations. Panels show measurements for (a) 10 mg/kg, (b) 25 mg/kg, (c) 50 mg/kg, (d) 75 mg/kg, and (e) 100 mg/kg PpIX. Dots represent the measured photon counts per time bin, and solid curves represent fitted decay profiles.

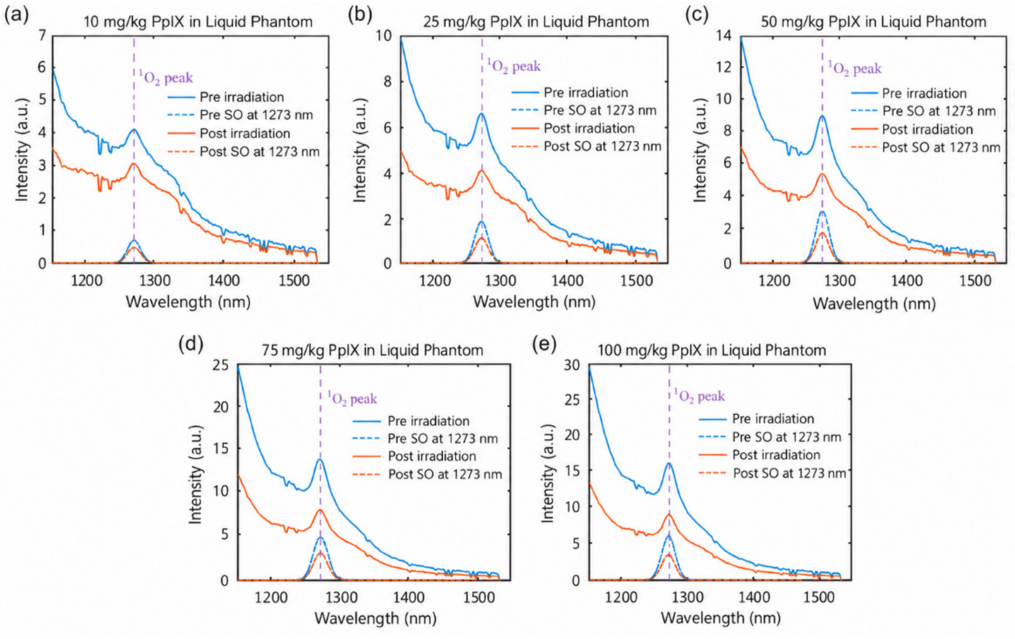

Figure S2. Raw MSOLD spectra at different PpIX concentrations. Raw multispectral singlet oxygen luminescence dosimetry (MSOLD) spectra acquired at the beginning and end of irradiation in liquid PpIX phantoms with different photosensitizer concentrations. Panels show spectra for (a) 10 mg/kg, (b) 25 mg/kg, (c) 50 mg/kg, (d) 75 mg/kg, and (e) 100 mg/kg PpIX. The spectra show near-infrared emission over the measured wavelength range, including the singlet oxygen luminescence region near 1270 nm.

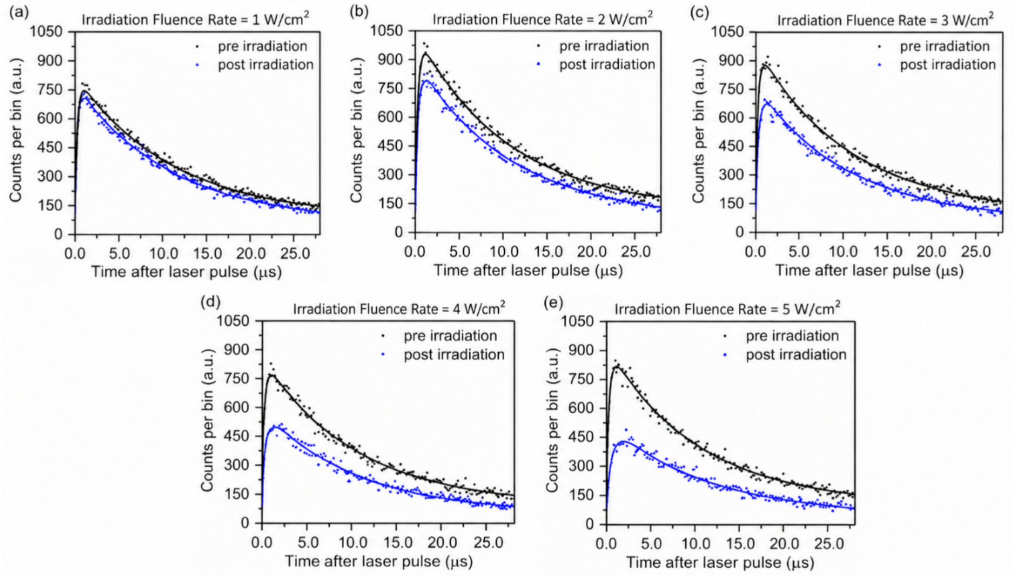

Figure S3. Raw TSOLD measurements at different irradiation fluence rates. Time-resolved singlet oxygen luminescence detection (TSOLD) photon-counting histograms acquired before and after irradiation at different fluence rates using a fixed PpIX concentration. Panels show measurements for (a) 1 W/cm², (b) 2 W/cm², (c) 3 W/cm², (d) 4 W/cm², and (e) 5 W/cm². Dots represent measured photon counts per time bin, and solid curves represent fitted decay profiles.

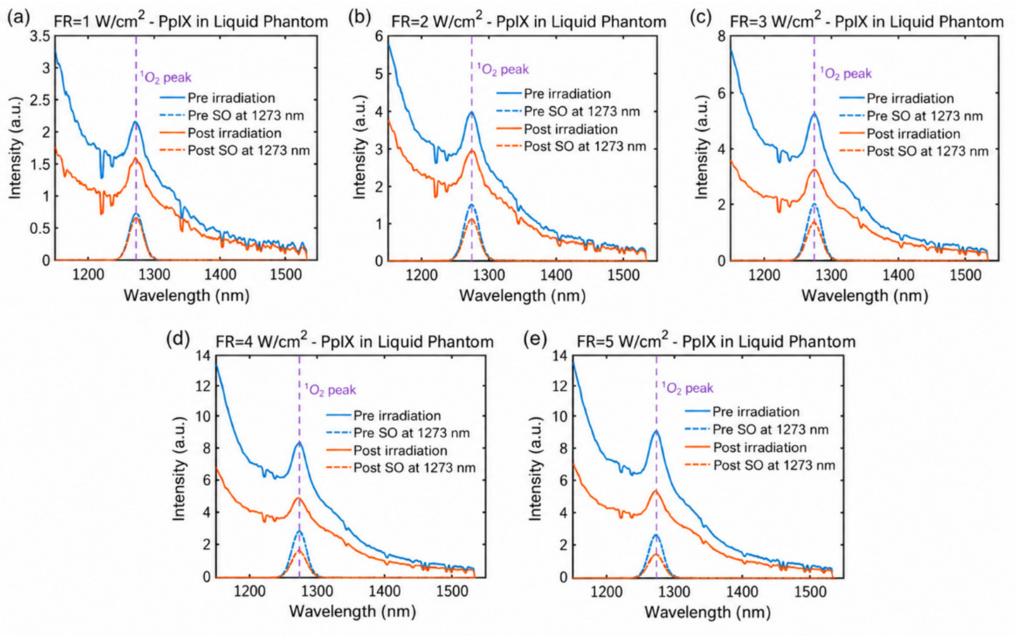

Figure S4. Raw MSOLD spectra at different irradiation fluence rates. Raw multispectral singlet oxygen luminescence dosimetry (MSOLD) spectra acquired at the beginning and end of irradiation in liquid PpIX phantoms at different fluence rates using a fixed PpIX concentration. Panels show spectra for (a) 1 W/cm², (b) 2 W/cm², (c) 3 W/cm², (d) 4 W/cm², and (e) 5 W/cm².

Figures S1–S4 present representative raw TSOLD and MSOLD measurements that support the quantitative analyses reported in the main manuscript. While the main text focuses on extracted percentage changes, fitted $^1O_2$ signals, and comparisons among TSOLD, and MSOLD, these supplemental figures show the original experimental data before further quantitative processing. For TSOLD, the photon-counting histograms demonstrate the time-resolved $^1O_2$ luminescence decay before and after irradiation. For MSOLD, the raw near-infrared spectra show the emission profiles acquired at the beginning and end of irradiation, including the spectral region around 1270 nm associated with $^1O_2$ luminescence.

Across both the concentration-dependent and fluence-rate-dependent experiments, the signals measured after or at the end of irradiation were lower than the corresponding initial signals, indicating an irradiation-induced reduction in measurable $^1O_2$ luminescence. This reduction may reflect photosensitizer photobleaching, transient changes in oxygen availability, thermal effects, or a combination of these factors. These figures demonstrate the observed signal reduction without assigning it to a specific underlying mechanism.

2. Data Tables for TSOLD and MSOLD Data Analysis

The A_{pre} and A_{post} values of Time-resolved photon histograms calculated by using bi-exponential curve fitting (Eq. 4 in the main manuscript) for different PpIX concentrations and irradiation fluence rates before and after irradiation are given in Table S1 and S2. The respective background values, A_0, are subtracted from the A values to estimate relative change in cumulative singlet oxygen for TSOLD measurements.

Table S1. Fitted amplitude (A) and background values obtained from histogram fitting at different PpIX concentrations under a constant fluence rate of 5 W/cm². These values correspond to the fits of the raw data shown in Figure S1. The uncertainties reported for A and background represent standard errors obtained from the bi-exponential fitting procedure.

**Conc. of PpIX (mg/kg)**	**Conc. of PpIX ($\mu mol/kg$)**	**A_{pre} Values**	**A_{post} Values**	**A_{pre} -A_{post} Values**	**Background A_0 values**
**10**	17.8	217 ± 1.6	173 ± 1.8	44 ± 1.7	20 ± 2.1
**25**	44.4	422 ± 0.5	248 ± 0.1	174 ± 0.2	20 ± 1.0
**50**	88.9	920 ± 2.3	527 ± 2.1	393 ± 2.2	20 ± 1.7
**75**	133.3	921 ± 0.8	454 ± 0.6	467 ± 0.6	20 ± 2.3
**100**	177.73	919 ± 0.8	465 ± 0.7	454 ± 0.5	20 ± 1.7

Table S2. Fitted amplitude (A) and background values obtained from histogram fitting at different irradiation fluence rates with a constant PpIX concentration of 50 mg/kg. These values correspond to the fits of the raw data shown in Figure S3. The uncertainties reported for A and background represent standard errors obtained from the bi-exponential fitting procedure.

**Irradiation Fluence Rate (W/cm^2)**	**A_{pre} Values**	**A_{post} Values**	**A_{pre} - A_{post} Values**	**Background A_0 values**
**1**	728 ± 0.3	701 ± 0.4	27 ± 0.3	20 ± 3.1
**2**	921 ± 0.8	804 ± 1.8	117 ± 1.1	20 ± 0.8
**3**	864 ± 3.1	674 ± 2.5	190 ± 2.7	20 ± 2.4
**4**	751 ± 0.5	506 ± 0.7	245 ± 0.6	20 ± 0.7
**5**	808 ± 0.5	444 ±0.9	364 ± 0.7	20 ± 0.4

Tables S3 and S4 summarize the PpIX concentrations measured both before and after 900 s of irradiation using a custom-built contact fluorescence probe. Table S3 presents the pre- and post-irradiation PpIX concentrations for experiments performed with different initial PpIX concentrations at a constant fluence rate of 5 W/cm², whereas Table S4 presents the corresponding pre- and post-irradiation PpIX concentrations for experiments performed at different fluence rates with the initial PpIX concentration held constant at 50 mg/kg. The decrease in PpIX concentration after irradiation reflects photosensitizer photobleaching during irradiation, and the reported uncertainties reflect variability among probe measurements at different sampling locations within the phantom.

Table S3. Nominal and measured PpIX concentrations before and after 900 s of irradiation at different nominal PpIX concentrations under a constant fluence rate of 5 W/cm². Reported uncertainties represent variability among repeated contact-probe measurements at different sampling locations within the phantom.

Nominal PpIX concentration (mg/kg)	Nominal PpIX concentration ($\mu mol/kg$)	Measured Conc. of PpIX_pre (μM)	Measured Conc. of PpIX_post (μM)
**10**	17.8	13.95 ± 0.04	8.08 ± 0.02
**25**	44.4	35.46 ± 0.38	26.39 ± 0.86
**50**	88.9	70.91 ± 2.07	59.67 ± 0.88
**75**	133.3	106.37 ± 2.03	82.60 ± 4.66
**100**	177.73	141.83 ± 8.67	110.05 ± 4.91

Table S4. Nominal and measured PpIX concentrations before and after 900 s of irradiation at different fluence rates under a constant nominal initial PpIX concentration of 50 mg/kg. Reported uncertainties represent variability among repeated contact-probe measurements at different sampling locations within the phantom.

Irradiation Fluence Rate (W/cm^2)	Nominal PpIX concentration ($\mu mol/kg$)	Measured Conc. of PpIX_pre (μM)	Measured Conc. of PpIX_post (μM)
**1**	88.9	71.55 ± 2.19	64.65 ± 1.06
**2**	88.9	70.20 ± 2.08	61.28 ± 1.23
**3**	88.9	70.66 ± 1.97	57.08 ± 0.90
**4**	88.9	70.33 ± 2.38	52.68 ± 0.97
**5**	88.9	71.29 ± 2.09	49.40 ± 0.71

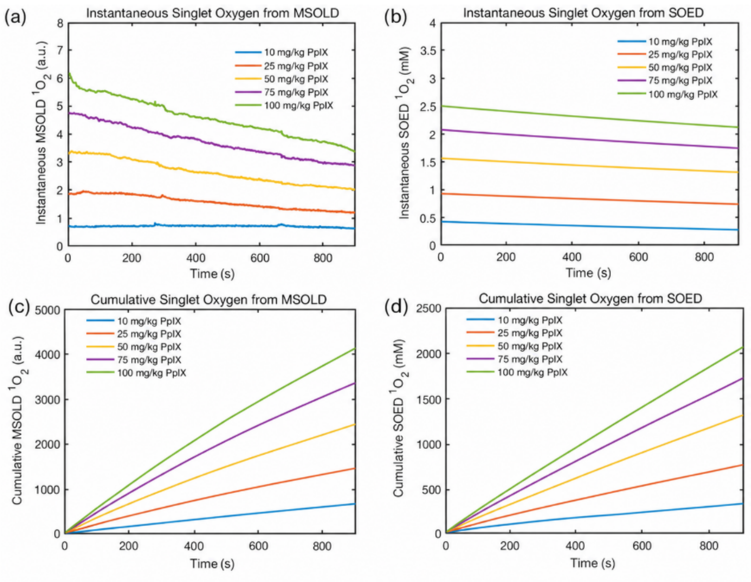

Figure S5. Comparison of instantaneous and cumulative $^1O_2$ signals measured by MSOLD and calculated by SOED at different PpIX concentrations. Instantaneous and cumulative singlet oxygen signals obtained from MSOLD measurements and SOED calculations for liquid PpIX phantoms with different photosensitizer concentrations. Panel (a) shows instantaneous MSOLD-derived $^1O_2$ signals in arbitrary units for PpIX concentrations of 10, 25, 50, 75 and 100 mg/kg PpIX; (b) shows the corresponding SOED-calculated instantaneous reacted $^1O_2$ concentrations in mM; (c) shows the corresponding cumulative MSOLD-derived $^1O_2$ signal in arbitrary units; (d) shows the corresponding SOED-calculated cumulative reacted $^1O_2$ concentrations in mM.

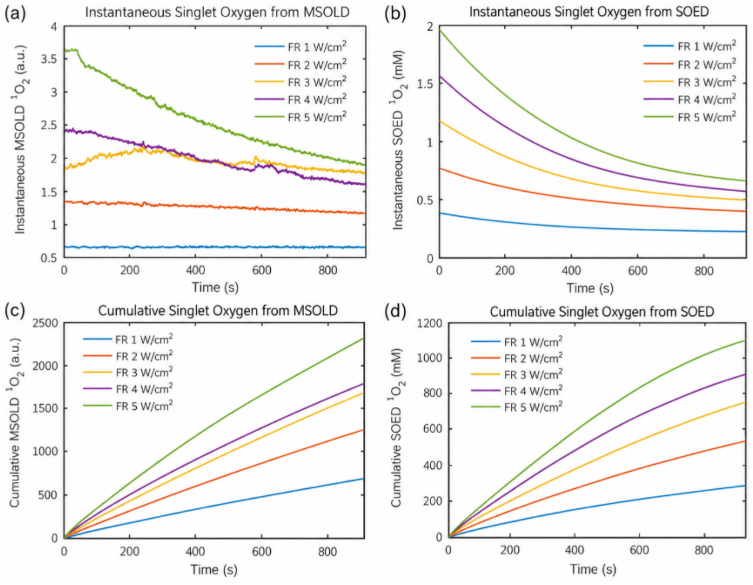

Figure S6. Comparison of instantaneous and cumulative $^1O_2$ signals measured by MSOLD and calculated by SOED at different irradiation fluence rates. Cumulative singlet oxygen signals obtained from MSOLD measurements and SOED calculations at different irradiation fluence rates using a fixed PpIX concentration. Panel (a) shows cumulative MSOLD-derived $^1O_2$ signals in arbitrary units for fluence rates of 1, 2, 3, 4, and 5 W/cm²; (b) shows the corresponding SOED-calculated instantaneous reacted $^1O_2$ concentrations in mM; (c) shows the corresponding cumulative MSOLD derived $^1O_2$ concentration in arbitrary units; (d) shows the corresponding SOED-calculated cumulative reacted $^1O_2$ concentrations in mM.

Supplementary Figures S5 and S6 provide the time-dependent instantaneous and cumulative $^1O_2$ signals used for the MSOLD–SOED comparison. Unlike the main-text correlation plots, which directly compare cumulative MSOLD and SOED values at selected time points, these supplemental figures show the original cumulative signal evolution as a function of irradiation time. The MSOLD-derived instantaneous and cumulative $^1O_2$ signal is reported in arbitrary units because it is obtained from spectral decomposition of measured luminescence signals, whereas SOED provides calculated reacted $^1O_2$ concentration in mM based on photophysical modelling. The cumulative MSOLD $^1O_2$ signal was calculated by numerical integration of the SVD-extracted instantaneous $^1O_2$ signal over irradiation time, using the effective acquisition interval of approximately 0.83 s per spectrum. Despite this difference in units, both approaches showed similar increases over time and similar dependence on PpIX concentration and fluence rate. In the fluence-rate-dependent comparison, the 3 W/cm^2 condition showed a different trend compared with the other fluence rates, which may be related to experimental variability in light delivery, probe positioning, or local oxygen availability. Because the overall monotonic increase with irradiation time was still preserved, this difference does not change the general agreement between MSOLD and SOED, but it highlights the sensitivity of the measurement to experimental geometry and local phantom conditions. These results provide visual support for the model-based comparison in the main manuscript, in which cumulative MSOLD signals were compared with SOED-calculated reacted $^1O_2$ under the defined experimental conditions.

3. PpIX Extinction Coefficient as a Function of Concentration

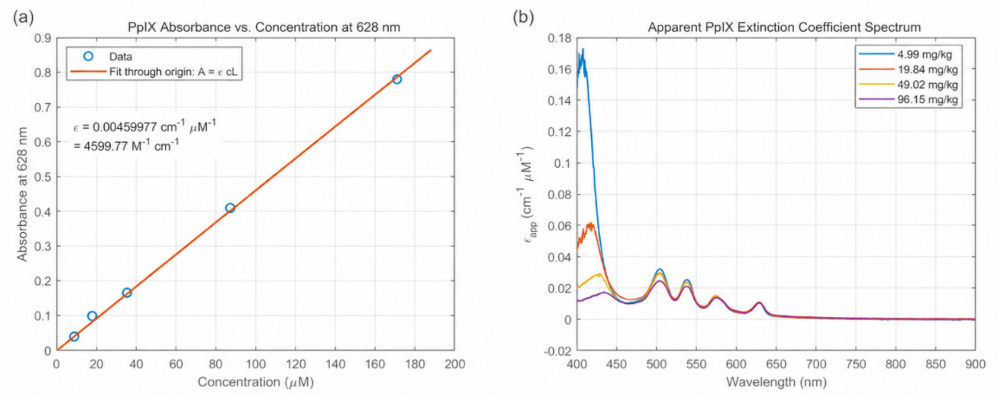

Figure S7. Experimental determination of the PpIX extinction coefficient in methanol. (a) Absorbance at 628 nm as a function of PpIX concentration. The data were fit through the origin according to the Beer–Lambert relationship, yielding an extinction coefficient of approximately 4599.8 $\mathrm{M}^{-1}\ \mathrm{cm}^{-1}$. (b) Apparent PpIX extinction coefficient spectra calculated from absorbance measurements at different PpIX concentrations.

As shown in Figure S7(a), absorbance at 628 nm increased linearly with PpIX concentration and was well described by the decadic Beer–Lambert relationship,

$$A_{10} = \varepsilon_{10} c l. \qquad \text{(S1)}$$

The fitted decadic extinction coefficient was approximately

$$\varepsilon_{10} = 0.0046\ \mathrm{cm}^{-1}\ \mu\mathrm{M}^{-1} = 4.6 \times 10^{3}\ \mathrm{M}^{-1}\ \mathrm{cm}^{-1}. \tag{S2}$$

For use in the exponential attenuation model

$$I = I_0 e^{-\mu_a l}, \tag{S3}$$

the decadic extinction coefficient was converted to the corresponding Napierian coefficient by multiplication by ln (10):

$$\varepsilon_e = \ln(10)\varepsilon_{10} = 2.3026 \times 0.0046 \approx 0.0106\ \mathrm{cm}^{-1}\ \mu\mathrm{M}^{-1}. \tag{S4}$$

No appreciable curvature or systematic deviation from linearity was observed at 628 nm over the investigated concentration range, supporting Beer–Lambert behavior under these conditions. In addition, Figure S7(b) shows that the apparent PpIX extinction coefficient spectra remained similar across the investigated concentrations, with no clear concentration-dependent spectral changes indicative of substantial PpIX aggregation.

4. Effect of Intralipid Scattering on TSOLD Signal

To evaluate the effect of optical scattering on the detected TSOLD signal, additional measurements were performed using PpIX at a nominal concentration of 100 mg/kg in pure methanol and in the methanol–Intralipid phantom containing 0.6% Intralipid. These measurements were performed using a separate 532 nm pulsed crystal laser (QL532-100-O, Crystalaser, Reno, NV, USA). According to the manufacturer, the crystal laser operates at a repetition rate of 20 kHz with a pulse width of approximately 18 ns FWHM and a nominal average output power of 45 mW. The optical power delivered through the experimental setup was approximately 3 mW. Photon-arrival histograms were acquired using a 15 ns bin width with a total acquisition time of 60 s. The excitation and acquisition settings were kept identical between the pure-methanol and methanol–Intralipid measurements.

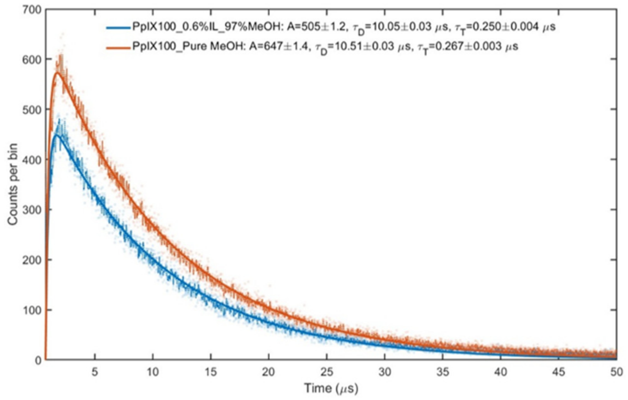

Figure S8. Comparison of TSOLD measurements in pure methanol and a methanol–Intralipid scattering phantom. Time-resolved singlet-oxygen luminescence measurements of PpIX at a nominal concentration of 100 mg/kg in pure methanol and in a methanol-based scattering phantom containing 0.6% Intralipid, using a 532 nm pulsed crystal laser operated at 20 kHz with a 20 ns pulse width and an average output power of approximately 3 mW. Dots represent measured photon counts and solid lines represent the corresponding fits. The fitted signal amplitude decreased from 647 ± 1.4 counts in pure methanol to 505 ± 1.2 counts in the methanol–Intralipid phantom, corresponding to an approximately 22% reduction. The fitted

singlet-oxygen lifetime decreased slightly from $10.51 \pm 0.03\ \mu s$ to $10.05 \pm 0.03\ \mu s$, while the fitted photosensitizer triplet-state lifetime changed from $0.267 \pm 0.03\ \mu s$ to $0.250 \pm 0.04\ \mu s$.

As shown in Figure S8, introduction of Intralipid reduced the detected TSOLD signal amplitude. The fitted amplitude decreased from 647 ± 1.4 counts in pure methanol to 505 ± 1.2 counts in the methanol–Intralipid phantom, corresponding to an approximately 22% reduction. In contrast, the fitted singlet-oxygen lifetime showed only a modest change, decreasing from in $10.51 \pm 0.03\ \mu s$ pure methanol to $10.05 \pm 0.03\ \mu s$ in the methanol–Intralipid phantom. The fitted photosensitizer triplet-state lifetimes were $0.267 \pm 0.03\ \mu s$ and $0.250 \pm 0.04\ \mu s$, respectively. The corresponding SNR values were 36.51 and 33.23. These measurements demonstrate that the introduction of optical scattering primarily reduces the detected TSOLD signal magnitude under the present experimental conditions, while producing relatively small changes in the fitted singlet-oxygen and photosensitizer triplet-state lifetimes.
